# Defining business model key performance indicators using intentional linguistic summaries

**DOI:** 10.1007/s10270-021-00894-x

**Published:** 2021-06-15

**Authors:** Rick Gilsing, Anna Wilbik, Paul Grefen, Oktay Turetken, Baris Ozkan, Onat Ege Adali, Frank Berkers

**Affiliations:** 1 Eindhoven University of Technology, Eindhoven, the Netherlands; 2 DKE, Maastricht University, Maastricht, the Netherlands; 3 Atos Digital Transformation Consulting, Eindhoven, the Netherlands; 4 TNO, Den Hague, the Netherlands

**Keywords:** Business model evaluation, Key performance indicators, Linguistic summarization, Intentional linguistic summaries, Business model innovation

## Abstract

To sustain competitiveness in contemporary, fast-paced markets, organizations increasingly focus on innovating their business models to enhance current value propositions or to explore novel sources of value creation. However, business model innovation is a complex task, characterized by shifting characteristics in terms of uncertainty, data availability and its impact on decision making. To cope with such challenges, business model evaluation is advocated to make sense of novel business models and to support decision making. Key performance indicators (KPIs) are frequently used in business model evaluation to structure the performance assessment of these models and to evaluate their strategic implications, in turn aiding business model decision making. However, given the shifting characteristics of the innovation process, the application and effectiveness of KPIs depend significantly on how such KPIs are defined. The techniques proposed in the existing literature typically generate or use quantitatively oriented KPIs, which are not well-suited for the early phases of the business model innovation process. Therefore, following a design science research methodology, we have developed a novel method for defining business model KPIs, taking into account the characteristics of the innovation process, offering holistic support toward decision making. Building on theory on linguistic summarization, we use a set of structured templates to define qualitative KPIs that are suitable to support early-phase decision making. In addition, we show how these KPIs can be gradually quantified to support later phases of the innovation process. We have evaluated our method by applying it in two real-life business cases, interviewing 13 industry experts to assess its utility.

## Introduction

As a result of factors such as globalization, rapid technology change and digitization, we observe that many contemporary markets become highly dynamic in nature and evolve at an accelerated pace [17, 37]. To sustain competitive advantage in such markets, many organizations focus on renewing or innovating their *business model*, to explore novel sources of value creation [41], to differentiate existing value propositions, or to avoid imitation by competitors [3]. A business model describes the logic of how an organization creates value for a customer (segment) and captures value in return [50], as well as details the resources and activities needed to do so [79]. Generally in management literature, such (value-focused) business models take the form of textual and visual representations [9], describing or illustrating how organizations collaborate, what business activities are conducted, what resources are deployed or exchanged and how value is created for the customer. Business models are considered to take a pivotal role for organizations, enabling organizations to translate strategic objectives into concrete business plans. As a result, business models often serve as the starting point for further deployment [2]. Not surprisingly, given its descriptive and structuring power, the business model concept has become increasingly prevalent in information systems research to explore how novel technologies can be contextualized or how they may contribute to value creation [68].

The innovation of business models, however, is considered to be a complex task or process [8], generally characterized by significant uncertainty with regard to business model decision making and its expected outcomes, particularly in the early phases of business model innovation [44]. To reduce such complexity and uncertainty, organizations can, in addition to practices of trial-and-error learning [60], strongly benefit from tooling and techniques directed at the *evaluation* of business models and structuring decision making [57, 68]. In response, research has paid ample attention to developing both qualitative [19–21, 43] and quantitative [7, 22] tools and techniques for the evaluation of business models, generating insights on the expected performance of business models, which in turn may support decision making.

Supporting business model evaluation, we observe the use of *key performance indicators* (KPIs) to further structure the performance assessment of business models [26]. KPIs represent measurable constructs that facilitate organizations to assess and monitor business performance relative to the objectives on which the KPIs have been built [42]. Generally, KPIs are defined based on business strategy, enabling organizations to translate abstract strategic objectives into concrete, measurable KPIs. In the context of business models, KPIs therefore are used to support business model decision making and to *evaluate* whether, or the degree to which, a business model design satisfies its strategic objectives [52].

However, the effectiveness of KPIs to support decision making depends significantly on the timing for which they are applied in the business model innovation process [74]. Business model innovation is an iterative process, for which a novel business model design is gradually developed and concretized over time. As a result, early phases of the innovation process are often characterized by significant uncertainty and limited data availability [44], even more so for business models that are new to the firm [15]. In such early phases, quantified KPIs offer limited support for decision making, as the performance of the business model design can hardly be accurately measured or predicted. Therefore, to support decision making, such KPIs should be catered to the characteristics of the innovation process, gradually quantifying as the business model design concretizes. However, examining current literature, we observe that research generally focuses on techniques toward the definition of quantitative (business model) KPIs [26, 33, 45]. As a result, these techniques offer limited guidance on the definition of qualitative KPIs suitable for use in early phases of business model innovation, as well as describing how these KPIs should be gradually quantified to support decision making. On the other hand, opinion-based techniques (such as expert judgment) as a means to account for qualitative decision making are often unstructured and do not offer much value toward measuring the performance of business models, particularly in later phases of the innovation process. As such, research lacks structured guidance for the definition of KPIs to support decision making throughout business model innovation, clarifying how KPIs can be defined and developed to cater to the characteristics of the innovation process. This is troublesome as limited decision support may result in poor or inadequately designed business models with limited long-term viability [55]. To address this research challenge, we pose the following research objective:

*To develop a method for the definition of business model key performance indicators (KPIs) catered to the characteristics of the business model innovation process to support business model decision making.*

Following a design science research methodology [51], we have iteratively developed a method as a design artifact. To cater to the shifting characteristics of the innovation process and to support the subsequent definition of KPIs, we draw upon theory on *linguistic summarization* for data summarization [75]. Whilst linguistic summarization is typically applied to make sense of data, its structure and properties can also be used to support decision making in business model evaluation: to generate linguistic (e.g., qualitative) summaries based on the (quantitative) intentions of stakeholders in the business model design. As such, these linguistic summaries are not inferred from data but rather capture the strategic goals or *intentions* of the stakeholders of a business model in a qualitative sense. These *intentional linguistic summaries* (ILSs) accordingly can be used as qualitative KPIs. Moreover, using the membership functions [76] underlying the ILSs, these ILSs can be gradually quantified during the business model innovation process as the business model is concretized, facilitating their use throughout the innovation process as a holistic support for informed decision making.

We have proposed an initial structure and formalization for this method in Gilsing et al. [19–21] and Wilbik et al. [72]. However, the initial version of the method lacked explicit guidance on its use and offered limited support for the gradual quantification of the ILSs or KPIs. Moreover, the initial method was only subjected to a preliminary evaluation. In the current paper, we have significantly extended the guidance on the use of the method, including gradual quantification of ILSs. We have also evaluated the validity and utility of the method through two real-life business scenarios, involving a group of 13 business experts to assess the use of the method in these scenarios.

Our research contributes to the ongoing call for decision making in business model innovation [55], by offering a method for the definition of KPIs that can be used throughout the innovation process, rather than supporting either qualitative or quantitative decision making. As a result, our method offers more holistic support toward decision making, contributing to the general understanding of how uncertainty with regard to business model design and innovation can be reduced. For practice, our method offers structured guidance on how qualitative KPIs can be defined to support decision making in early phases of business model innovation, and how these KPIs can be gradually quantified to support decision making in later phases of this process.

The remainder of this paper is structured as follows: In Sect. 2, we present the literature background for our work and discuss the related work on business model evaluation. Next in Sect. 3, we discuss the research design we have followed to develop our method. In Sect. 4, we elaborate on our method, describing how the method supports the definition of KPIs for business model innovation and how the techniques that are embedded within the method are used. In Sect. 5, we discuss the results of the first design cycle of our method, for which we evaluate the alpha version of the method. Next, in Sect. 6, we elaborate on the evaluation of the beta version of our method based on the feedback received. We conclude our paper in Sect. 7 by listing the main contributions of our method, the limitations to our work and avenues for future research.

## Literature background and related work

In this section, we discuss the literature background and related work for our research. First, we discuss the background on business model design, where we elaborate on the SDBM/R technique, which we use to represent business models in the application of our method. Next, we discuss the concept of business model innovation and how its characteristics influence decision making. We also elaborate on the existing work with respect to business model evaluation and how KPIs are used to complement this decision making. Finally, we discuss the background of linguistic summarization with regard to its role as a technique in our method.

### Business model design

To represent and support the exploration of business models, research has focused on the development of business model design tools [9]. As a result, several tools have been proposed for the design of business models. Widely popular amongst both practitioners and researchers, Osterwalder and Pigneur [50] propose the business model canvas (BMC). The BMC technique uses a graphical template consisting of nine building blocks that together make up a business model design for a specific organization. These building blocks represent key business model elements, such as the value proposition to customers, the resources deployed, and the business activities conducted to create and capture value. BMC reflects the perspective of a single organization and explains how it aims to create and capture value.

In light of an increased service-orientation amongst organizations [35] and the rise of collaborative networks [10], we observe that research has also focused on the development of networked-oriented, service-driven business model design tooling to cater to the need of more contemporary business initiatives. For instance, Zolnowski et al. [78] propose the service business model canvas (SBMCs), which represents a ‘stack of BMCs’ to accommodate a networked perspective of business models. Each individual BMC is adapted to accommodate the concepts related to service-dominant business or service provisioning. Similarly, Grefen [23] and Turetken et al. [66, 67] propose the service-dominant business model radar (SDBM/R) to represent service-dominant business models. In contrast to the SBMC, the SDBM/R represents a circular template with a central value-in-use at its core, which is co-created through the activities and resulting value propositions of the network of actors that surrounds this core. As a result, the SDBM/R facilitates the explicit modeling of how value is co-created through service provisioning in networked collaborations (rather than a bundled stack of organizations). Given its explicit capability to represent contemporary networked business models, we use the SDBM/R for the remainder of this work to represent business models and as a basis to our method.

#### Service-dominant business model radar (SDBM/R)

**Fig. 1:**
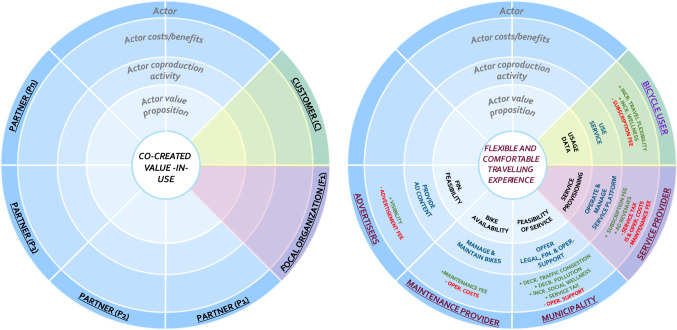
SDBM/R template for business model design (left) and example business model for bikesharing (right)

The template for the SDBM/R technique is presented in Fig. 1 (left). SDBM/R takes the value-in-use (i.e., the value to-be created for the customer) at its core and is divided into ‘pie slices’ that surround this core. These pie slices represent actors that are part of the business network and participate for and contribute to the respective business model design (represented by the outer ring). The SDBM/R contains three rings that for each actor (in its respective pie slice) describe the *actor value proposition* (the value that is proposed by an actor as part of value-in-use), the *actor co-production activities* (the activities conducted to establish the value proposition), and the *actor costs and benefits* (the respective costs and benefits per actor that are expected to result from participating in the business model execution).

With regard to the set of actors, each *service-dominant* business model design should feature at least one customer (or customer segment) to which the value-in-use is directed, one focal organization or orchestrator, and at least one other business network actor (to account for a networked business model perspective). The set of business network actors can be further classified as either *core parties* (e.g., are essential for the execution of the business model or the delivery of the service solution) or *enriching parties* (e.g., extend the value proposition or enrich the service offering).

An example of a completed business model design can be seen in Fig. 1 (right). One can see that the business model focuses on the value-in-use ‘Flexible and comfortable travelling experience’, and features roles such as the user, service provider, municipality, maintenance provider and advertisers (each with associated value propositions, activities and costs and benefits) to describe the logic by which the business model is expected to create value and how this is to be supported.

The SDBM/R has been successfully applied in a set of industry projects to represent networked business models, for which the results on its application and evaluation have been communicated with scholars in a number of publications [1, 6, 18, 23, 24, 38, 62, 65–67].

### Business model innovation and decision making

Business model innovation (BMI) has been defined as “the process of designing a new or modifying the firm’s business model or the discovery of fundamentally different business models” [40, 79]. It is therefore not surprising that BMI is often considered as an iterative process, featuring sequential steps or phases that guide business models from ideation toward implementation. To support the BMI process, several scholars have investigated the activities that are typically conducted toward the innovation of business models [74]. Generally, business model innovation is preceded by an exploration phase for which organizations analyze the strategic goals or objectives to be pursued, justifying the need for novel business model design or business model redesign. As per the start of the innovation process, novel business model designs are ideated, taking into account the strategic challenges posed. Depending on the preliminary performance of these business model designs, model alternatives are either discarded or selected for further concretization, which constitutes the next step of the innovation process. This concretization step is iterative, for which the goal is to find a concrete business model design (in terms of its structure and underlying business case) that works for all involved stakeholders. Once the business model design is complete, the innovation process is concluded by an implementation step, focusing on the implementation of the model into the logic of the organization(s).

The BMI process inherently is uncertain and generates ambiguity and risk for decision making [55]. This is particularly relevant for new-to-the-firm business models, which concerns finding a viable novel business model design without any preconceived notion of whether the business model design may work in practice [56]. Particularly in early phases of the innovation process, data availability is generally low or lacking accuracy [44], rendering the use of qualitative decision making more appropriate [64]. However, as business models progress through the innovation phases, they become increasingly structured as decisions are finalized, facilitating the application of more quantitatively-oriented evaluation techniques [44]. To support decision making in business model innovation, practices of experimentation and trial-and-error learning are frequently stressed [5, 60]. In addition, business model evaluation is advocated as a means to structure decision making and to clarify the performance of business models [57].

### Key performance indicators for business model evaluation

Business model evaluation is defined as the act of analyzing and understanding the (perceived) performance of a business model design [44]. Through business model evaluation, decision makers are able to reduce uncertainty and risk by clarifying how design decisions may impact business model performance as well as facilitating the comparison between different business model alternatives [7]. Not surprisingly, business model evaluation is argued to positively influence business model innovation and innovation success [57]. To structure and support business model evaluation, several tools and techniques have been proposed, which can be divided into qualitative-oriented techniques based on criteria analysis, elicitation techniques or expert opinions [13, 19–21, 43] and quantitatively oriented support based on simulation modeling and financial metrics [7, 19–22, 45]. These tools enable decision makers analyze business models and to generate insights on their expected performance.

Complementing business model evaluation, we observe the use of key performance indicators (KPIs) for business models to structure the performance assessment of business models and to better understand its strategic implications [26, 52]. Several works have focused on proposing techniques for the definition of KPIs to support decision making. For instance, the popular Balanced Scorecard [33] is frequently used to translate the strategic objectives for a business model design into a concrete set of measures that can be used to evaluate the business model design. Similarly, Heikkila et al. [26] propose a repository of performance metrics or KPIs that can be used by business modelers to offer further insights on the expected performance of a business model design and are grouped based on what concern of a business model they address (e.g., related to the offering of the business model, its customer or the business processes supporting it). Closely related, Sharma and Gutierrez [58] propose a viability framework for m-commerce business models expressing critical success factors or performance indicators related to the design and performance of such business models.

However, we observe that these techniques generally focus on the definition or use of quantitatively oriented KPIs. Given the characteristics of the innovation process, for which it is difficult to quantify or accurately predict the performance of a business model early on [44], such quantitatively oriented KPIs are not well-suited for early-phase business model innovation. Accordingly, such KPIs should be adapted (in a qualitative sense) to accommodate early-phase decision making. However, the available techniques offer limited support with respect to how KPIs can be expressed in a qualitative sense to offer support toward qualitative, early-phase decision making and how these can gradually be quantified. On the other hand, techniques based on the elicitation of opinions [16], such as the expert reviews, offer only partial support toward quantitative decision making in the late phases of the innovation process. In addition, such techniques lack formal structure with respect to how performance indicators should be specified. Contrastingly, goal-setting techniques such as SMART goals offer structure in terms of how qualitative goals or performance directives should be specified, but are not well-catered to business model design and modeling. Hence, research lacks a holistic support (i.e., *throughout* the business model innovation process) to support the specification and definition of business model KPIs, catered to the characteristics of the business model innovation process. Such a method should guide the specification of qualitatively oriented KPIs that can be gradually quantified to support decision making in different phases of the innovation process.

### Linguistic summaries

As we mentioned in the introduction, we have adopted the intentional linguistic summaries (ILSs) in the qualitative and structured definition of KPIs in order to accommodate the shifting characteristics of the business model innovation process.

Linguistic summarization is a technique that is rooted in fuzzy set theory [34] with the aim to capture uncertainty. The core notion is a fuzzy set with boundaries that are not precise [76]. The membership in a fuzzy set is not a binary relation, but a matter to a degree. Many notions are measured on a sliding scale, such as a new car, successful business, tall person. Such a sliding scale often makes it impossible to distinguish members of a class from non-members [48]. For instance, for a new car, we could draw an arbitrary threshold of five days old, but it would mean that a car that is six days old is not new. Fuzzy logic solves such inconsistencies with human understanding allowing vagueness and a gradual transition between notions. Below, we present the basic concepts and definitions related to fuzzy sets [28, 34, 48].

A fuzzy set *A* in a universe of discourse *X* = {*x*}, written *A* in *X*, is defined as a set of pairs:

$$ A = \left\{ {\left( {\mu_{A} \left( x \right),x} \right)} \right\} $$

where *μ*_*A*_:*X* → [0, 1] is the membership function of *A* and *μ*_*A*_(*x*) ∈ [0, 1] is the grade of membership (or a membership grade) of an element *x* ∈ *X* in a fuzzy set *A*. A fuzzy set *A* is said to be empty, written *A* = ∅, if and only if *μ*_*A*_(*x*) = 0, for each *x* ∈ *X.*

The support of a fuzzy set *A* in *X*, written supp_A_, is the following (nonfuzzy) set:

$$ {\text{supp}}_{{\text{A}}} = \left\{ {x \in X:\mu_{A} \left( x \right) > 0} \right\}\;{\text{and}},\;{\text{evidently}},\;\emptyset \subseteq {\text{supp}}_{{\text{A}}} \subseteq X. $$

The core of a fuzzy set *A* in *X*, written core_A_, is the following (nonfuzzy) set:

$$ {\text{core}}_{{\text{A}}} = \left\{ {x \in X:\mu_{A} \left( x \right) = 1} \right\} $$

A nonfuzzy cardinality of a fuzzy set *A* = *μ*_*A*_(*x*_1_)/*x*_1_ + ··· + *μ*_*A*_(*x*_*n*_)/*x*_*n*_, the so-called sigma-count, denoted Σ-Count(A), is defined as [76, 77]:

$$ \Sigma {\text{ - Count}}\left( A \right) = \sum i = 1 \ldots n\;\mu_{A} \left( {x_{i} } \right). $$

The complement (corresponding to the negation ‘not’) of a fuzzy set *A* in *X*, written ¬*A*, is defined as:

$$ \mu_{\neg A} \left( x \right) = 1 - \mu_{A} \left( x \right),\;{\text{for}}\;{\text{each}}\;x \in X $$

The intersection of two fuzzy sets *A* and *B* in *X*, written *A* ∩ *B*, is defined as:

$$ \mu_{A \cap B} \left( x \right) = \mu_{A} \left( x \right) \wedge \mu_{B} \left( x \right),\;{\text{for}}\;{\text{each}}\;x \in X $$

where “∧” is the minimum operation, i.e., *a* ∧ *b* = min(*a*, *b*); the intersection of two fuzzy sets corresponds to the connective “and”.

The union of two fuzzy sets *A* and *B* in *X*, written *A* + *B*, is defined as:

$$ \mu_{A + B} \left( x \right) = \mu_{A} \left( x \right) \vee \mu_{B} \left( x \right),\;{\text{for}}\;{\text{each}}\;x \in X $$

where “∨” is the maximum operation, i.e., *a* ∨ *b* = max(*a*, *b*); the union of two fuzzy sets corresponds to the connective “or”.

Fuzzy sets have the same fundamental properties as crisp sets, e.g., associativity, commutativity, distributivity, de Morgan Laws, idempotency, identity, involution and transitivity.

**Fig. 2:**
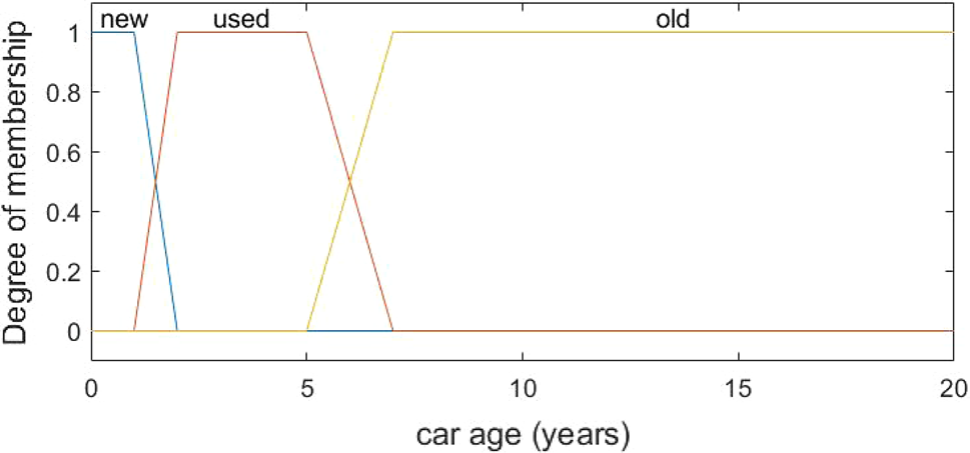
Example of fuzzy membership

Linguistic summaries employ this theory, especially the concepts such as linguistic variable and linguistic value. In the example of a new car, age of the car is a linguistic variable, and it can be described by three linguistic values: new, used, old. Each of the linguistic values is represented as a fuzzy set (Fig. 2).

In this paper, we use the approach to linguistic summarization of databases proposed by Yager [75]. Although this approach was originally proposed to be applied to numerical data (databases) [11, 27, 28, 31, 32, 59], later it was extended and adapted to other types of data, such as time series [29, 47], sensor activation data [53, 73], standardized texts [63], videos [4] and process data described in event logs [14, 71].

Linguistic summaries are automatically generated natural-language-like sentences expressed as quantified propositions generated from data. The original proposition for linguistic summarization [75] includes two protoforms (or templates):

- Simple protoform:
  1 $$ Q y^{\prime}s \;{\text{are}}\;P $$
  e.g., most cars are new
- Extended, qualified protoform
  2 $$ Q R y^{\prime}s\;{\text{are}}\;P $$
  e.g., most new cars are fast
  where *Q* denotes the quantifier, *P* is the summarizer, and *R* is an optional qualifier. These elements are all linguistic variables, modeled as fuzzy sets over appropriate domains. They are defined by the users, so that the linguistic labels match with the users’ mental model of those words.

All work on linguistic summaries concentrates on using these summaries to describe existing data, i.e., states or events in the past. We propose the use of linguistic summarization to structure the translation of quantified intentions of business model stakeholders into qualitative statements, facilitating such statements to be used as KPIs for early phases of the innovation process. Using the underlying membership functions for the linguistic variables set, these statements can be applied and gradually refined throughout the business model innovation process.

## Research design

**Fig. 3:**
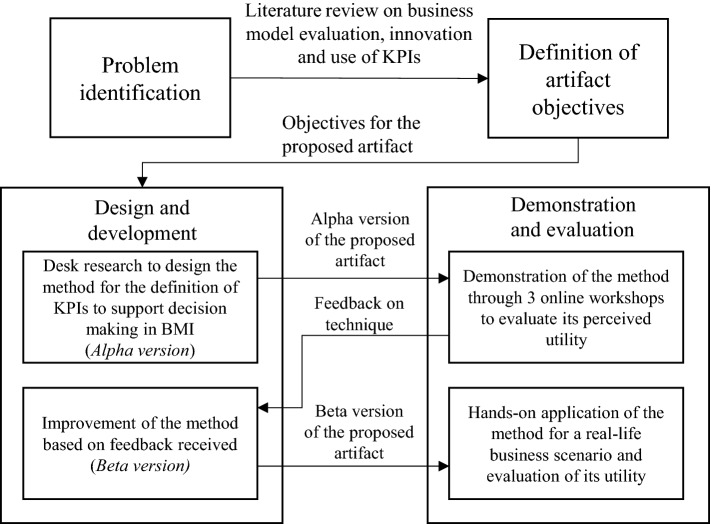
Research design

To guide our research endeavor, we have followed a design science research methodology [25]. In doing so, we identify the following research steps [51], as also illustrated in Fig. 3: problem identification, definition of artifact objectives, design of the artifact, demonstration and evaluation of the artifact. In the subsequent sections, we briefly detail each of the four steps.

### Problem identification

We have highlighted the problem to our research in Sects. 1 and 2 of this paper. To summarize, in supporting business model innovation, practices of business model evaluation are generally advocated to support decision making on the design and concretization of business models. We observe that KPIs are frequently used to complement business model evaluation, enabling stakeholders to better understand the performance of business models and to verify whether a business model design caters to the strategic needs of the respective stakeholder. However, to be adequately applied for the innovation process, which entails varying degrees of uncertainty and poses different requirements in terms of decision making throughout the process, careful attention should be paid toward the definition of KPIs. However, the definition of KPIs is often considered only from a quantitative perspective, making them ill-suited for the early phases of the business model innovation process. On the other hand, techniques such as expert judgment lack structure and offer limited support toward later phases. As a result, a structured and holistic support is lacking in research for the definition of business model KPIs that cater to the characteristics of the innovation process. As a consequence, innovated business models may suffer from uninformed or poor design decisions, which may threaten their long-term success and viability. Our proposed method aims to address this challenge.

### Definition of artifact objectives

Given the identified research problem, we define the following two objectives that should be satisfied by our proposed method:

#### Objective 1

*The proposed method should enable decision makers to define business model KPIs applicable for early-phase decision making in the innovation process.*

*Rationale*: Although KPIs are useful in structuring and supporting the performance assessment of business models and understanding whether a business model design satisfies its predetermined strategic objectives, they generally are quantitatively oriented. This makes it difficult to employ them in the early-phase decision making. To offer a holistic support toward decision making for business model innovation, our method therefore should enable decision makers to, in a structured way, define qualitatively oriented KPIs, such that these can be used in early phases of the innovation process.

#### Objective 2

*The proposed artifact should support decision makers in gradually quantifying business model KPIs to account for decision making in late phases of the innovation process.*

*Rationale*: To account for decision making in late phases of the innovation process, the qualitatively oriented or soft-KPIs should be gradually quantified, taking into account characteristics, such as business model uncertainty and accuracy of data. To offer a holistic support toward decision making in business model innovation, our method should accommodate users to do so.

### First design and evaluation cycle

For developing our artifact, we conducted two rounds of design and development complemented by two rounds of evaluation. For the first design and development cycle, we conducted a desk research on theory with respect to business model innovation, evaluation and the use of KPIs, and theory on linguistic summarization to iteratively develop the alpha version of the proposed method. In addition to the theory, we built upon the previous business model workshops that we conducted, which focused on conceptualizing business initiatives through business modeling. We drew on the challenges that workshop participants faced when using KPIs to complement business model evaluation. Any deficiencies identified with respect to the use of KPIs in these settings served as further input for the development of our method. For example, we observed that during business model design workshops, participants frequently focused on quantifying elements of the business model design (such as the expected number of customers) and setting quantified KPIs that reflect this. However, given the often abstract nature of business model designs at early phases of the innovation process, such KPIs were consequently difficult to use as the performance cannot yet be accurately assessed.

For the development process at least three researchers were involved with significant experience in the domain of business model design and evaluation, whereas one researcher had significant experience on the use and application of linguistic summarization. This alpha version of the method was subjected to a business scenario drawn from practice, after which we assessed whether the objectives set for the method were satisfied (validity of the method) as well as gain insights on the perceived utility generated through use of the method (utility of the method) [25]. The business scenario featured in a mobility setting in which stakeholders sought after a collaborative solution (through business modeling) to address mobility problems. This scenario is further detailed in Sect. 5. To understand the utility of the method, we organized three online workshops, featuring 11 industry experts, in which we demonstrated the use of the method for the aforementioned business scenario and consequently evaluated the *perceived usefulness*, *perceived ease-of-use* and *perceived intention-to-use* [70] with the industry experts. Any additional feedback was used to further improve the method.

### Second design and evaluation cycle

Based on the feedback received, we conducted a second design and development cycle. Specifically, with respect to *objective 2*, we more explicitly clarified how KPIs can be quantified throughout the business model innovation process using the underlying membership functions for the included linguistic variables. Moreover, in relation to *objective 1*, we further refined the steps taken to translate strategic objectives into business model-catered KPIs in order to improve the ease-of-use of the method. Similar to the first design and evaluation cycle, four researchers were involved. The resulting *beta version* of the method was consequently applied to a real-life business case, which focused on the application of the method by the stakeholders to support business model decision making. This real-life business case featured in a horticultural setting, for which a collaboration of stakeholders explored through business modeling efforts how a novel service aimed at improving decision making for growers could be offered and marketed. For this case, two business experts (both acting as project managers) were involved for the hands-on application of the method, defining soft-KPIs for the stakeholder roles represented for the associated business model design, and to explore how such KPIs can be further quantified. To evaluate the resulting utility, we conducted semistructured interviews with both business experts after the application of the method. The second design and evaluation cycle are further detailed in Sect. 6.

## Method description

**Fig. 4:**
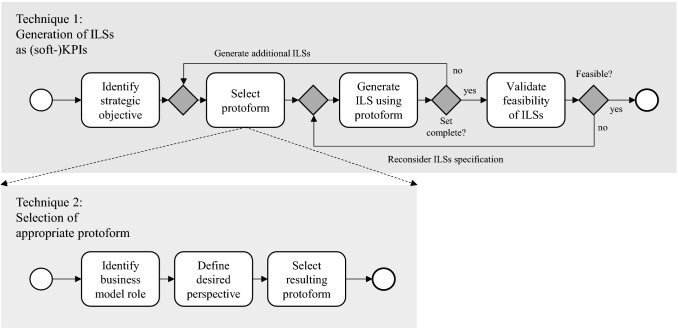
Method overview

In this section, we describe the working of our method. Our method is composed of two techniques that are used in an iterative fashion for the definition of ILS to support business model decision making, which are based on strategic objectives and can be used as KPIs. An overview of the method is presented in Fig. 4. The first technique (*technique-1*) involves the generation of ILSs as (soft-)KPIs. Regarding technique-1, Sect. 4.1 discusses the steps taken to define ILSs based on a strategic objective. To provide further guidance for the definition of appropriate ILSs (specifically the selection of protoforms as a base for their definition), we leverage *technique-2*, which is further described in Sect. 4.2. This technique details a catalogue of protoforms, catered to service-dominant business models. It offers a structure with regard to selecting appropriate protoforms, serving as *descriptive* suggestions for the definition of KPIs. Lastly, in Sect. 4.3, we explain how both techniques are integrated to constitute our method and describe how the method can be used to support decision making in business model innovation.

### Technique-1: generation of ILSs as (soft-)KPIs to support decision making

Technique-1 employs linguistic summarization to support the definition of so-called ILSs that may serve as KPIs to support decision making. As described in Sect. 2.4, both a simple (1) and extended (2) protoforms are defined for linguistic summarization to describe or summarize quantitative insights for a specific dataset into qualitative statements in a structured way. Depending on the quantifiers, qualifiers or summarizers selected per statement, users can account for any uncertainty or variance that is present for the dataset.

For instance, through varying the quantifier of any protoform, users can indicate how frequent a certain statement is expected to be true in qualitative terms. The advantage of this is that users are not burdened with the (accurate) quantification of how often this is expected to be true, but rather can incorporate degrees of freedom to interpret each statement. We draw upon this property for our method, summarizing the quantitative goals stakeholders may have with respect to business model performance into qualitative statements. Accordingly, using linguistic summarization, we define (soft-)KPIs that possess ample degrees of flexibility, suitable for use in early phases of business model innovation.

**Fig. 5:**
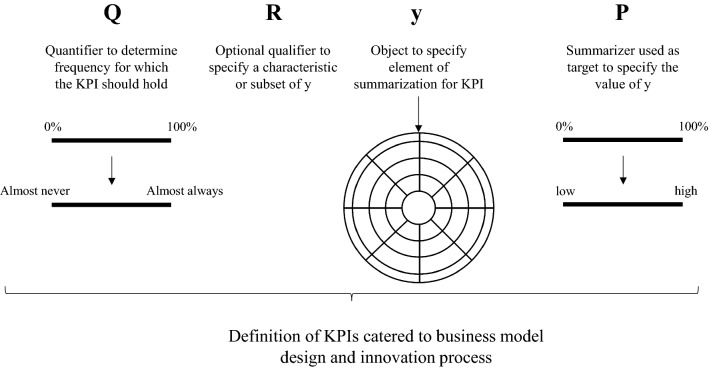
Using protoforms to define ILSs/KPIs to support decision making in business model innovation

For defining KPIs, the generic protoforms can be used as follows (as also depicted in Fig. 5):

1. First, a user determines what strategic objectives it desires to achieve with respect to the business model design and its expected performance. Based on these strategic objectives, the element *y* for the protoforms is used to select an element of the business model design (such as a cost, benefit, an activity, role or value) that would facilitate the translation of the strategic objective into one or more KPIs, for which *y* serve as the object of interest or summarization. To connect this to the SDBM/R technique, the set of elements selected is bounded to the elements represented for the SDBM/R. Note that, for the extended protoform, *R* can be used to further specify or characterize the selected (set of) element(s).
2. Next, a user specifies, using the summarizer *P*, what value or characteristic the element *y* should possess (dependent on the strategic objectives of the users), which corresponds to the target typically set for KPIs (e.g., a ROI of 10%). To cater such KPIs to early phases of the innovation process, quantitative intentions should be expressed in qualitative terms (e.g., the ROI should be *high*). Note here that the terms to be selected depend on the perceptions of the stakeholder defining the respective KPI. For example, some stakeholders may deem 10% as *very high*, whereas others may consider this to be *high* or even *moderate*.
3. Lastly, using the quantifier *Q*, the user indicates the frequency for which the KPI should be true or hold, incorporating additional flexibility of use if so desired. Again, to be used in the early phases of the business model innovation process, this quantifier should ideally be expressed in qualitative terms (e.g., *most of the times* or *for almost all*).

Following the indicated steps and building upon the generic protoforms, each user can express KPIs catered to a specific business model design that can be used for business model evaluation.

#### Quantification of Soft-KPIs

As business model designs advance and concretize through the innovation process, data to assess the expected performance or outcomes of a business model will gradually become more available and accurate [44]. As a consequence, to further support decision making, the initial KPIs should be gradually quantified using the fuzzy sets underlying the linguistic summarizers defined per KPI.

**Fig. 6:**
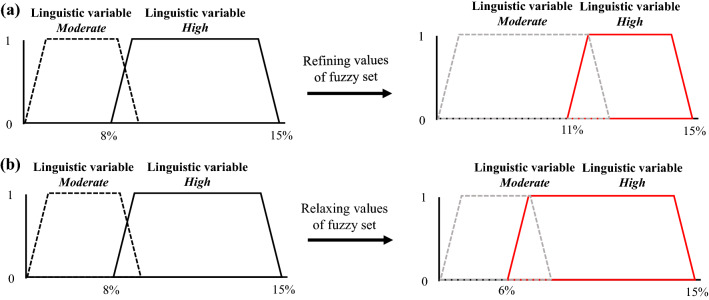
Gradual quantification of linguistic summarizers for KPIs

Referring back to the example KPI with respect to achieving a *high* ROI, for something to be classified as *high* the boundaries of the fuzzy set may initially be considered to be between 8 and 15% (see Fig. 6). As the business model design concretizes, these boundaries can be further refined depending on the expected outcomes of a business model design and the strategic motives or desires of the respective stakeholder. For instance, the expected performance of a business model design may be more than expected, leading stakeholders to refine (in quantitative terms) what *high* corresponds to (see Fig. 6a). On the other hand, if the performance is less than the expected, stakeholders can consider to relax what values classify as *high*, adjusting the core of the membership function accordingly (see Fig. 6b). Note that both the slopes of the membership function (for which the membership function is between 0 and 1, indicating that other linguistic variables may also apply for a given value) and the core of the membership function (membership function is 1, meaning only a single linguistic variable can apply) can be adjusted, depending on the perception or intentions of the respective stakeholder.

### Technique-2: selection of appropriate protoforms for definition of KPIs

Although the initial protoforms can be used to define KPIs, these protoforms are generic and as such offer limited guidance, particularly in the context of a business model design method (such as the SDBM/R). As part of our method, we propose a set of protoforms as *descriptive* suggestions (rather than *prescriptive* constraints) to further support the definition of KPIs.

In our previous work [19–21, 72], we have proposed an initial set of protoforms to generate KPIs. In this work, we further extend this set to accommodate different roles (such as the customer, orchestrator or global perspective) that can be present for (service-dominant) business models (constituting Technique-2). An overview of the proposed protoforms per different roles is presented in Table 1. We have defined four perspectives corresponding to four generic roles that feature in service-dominant business models. For marked combinations of perspective and role, more specific protoforms are defined to further support the definition of ILSs to be used as KPIs.

**Table 1:** Overview of the protoforms for different roles and perspectives

	Party-related ILS			Network-related ILS
	Customer-related ILS	Orchestrator-related ILS	Other party-related ILS	
Value-in-use perspective	*X*			
Transaction perspective	*X*	*X*	*X*	
Single-aggregated perspective		*X*		*X*
Double-aggregated perspective		*X*		*X*

In the next subsections, we first introduce a running case that is used to illustrate the working of the protoforms (Sect. 4.2.1). Next, in Sects. 4.2.2–4.2.5. we detail the extended set of protoforms and illustrate their working using this running case.

#### Illustrative business model case

**Fig. 7:**
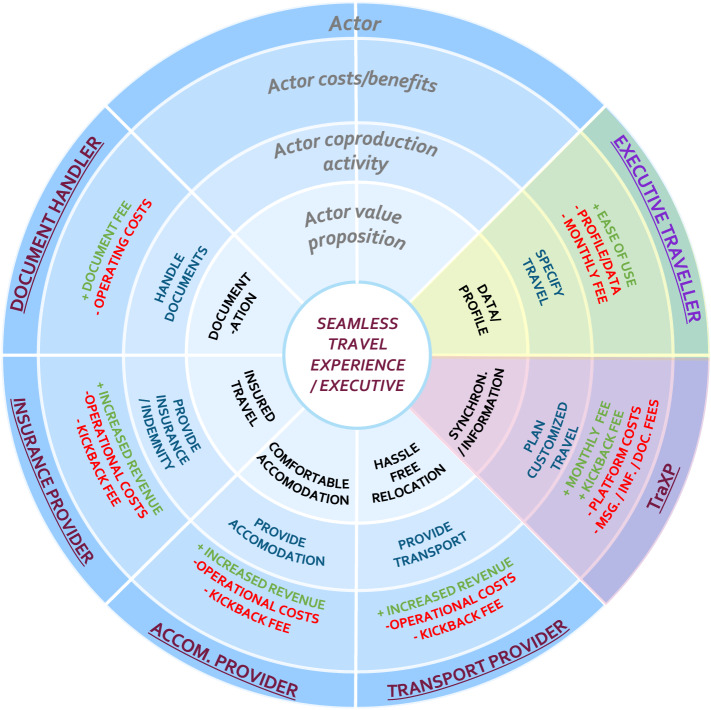
Service-dominant business model for 'seamless travel experience'

The business case that we use to illustrate our method focuses on offering a seamless travel experience to specific traveler types (customer segments) from a service-dominant business perspective [23, 24]. Depending on the type of customer segment, a complete travel solution constitutes the orchestration of the activities of a set of business partners that offer concurrent services, such as accommodation providers, taxi services, and airline service providers. A seamless travel experience refers to a service where the customer does not have to put in any effort for traveling other than being at the correct place at the right time. Such a business collaboration can be represented by means of a service-dominant business model (as illustrated in Fig. 7).

In this business model, a *focal organization* (referred to in the business model design as TraXP) offers a seamless travel experience to its executive travelers (the *customer* segment). To do so, the activities of several service providers, such as the transport providers (e.g., airlines, taxi’s), accommodation providers (hotels), insurance providers and document handlers, are integrated such that the executive traveler receives a complete coherent solution (e.g., seamless travel experience). Given the proposed solution, the accommodation and transport providers are considered as *core parties* (constituting the essence of any trip), while the insurance provider and document handler are considered *enriching parties* (enhancing the overall value proposition).

In the following, we detail the protoforms, which we list per the respective roles they address for service-dominant business models (e.g., the customer, orchestrator, party or business network role).

#### Customer-related protoforms

For the generation of KPIs (in the form of ILSs) with respect to the *customer* role, we focus on the *costs* and *benefits* indicated for the customer, as well as the *value-in-use* of the corresponding SDBM/R design. Therefore, ILSs generated to support business model evaluation from the customer perspective focus on those aspects of the business model design. Based on these templates, a customer can select objects and features that are most appropriate to express his or her individual strategic goals or motivations to participate.

##### Value-in-use perspective

The ILS of the *value-in-use* related to the customer role of the business model states that the majority of customers receive the value-in-use:

3 $$ {\text{In}}\;Q\;{\text{transactions}}\;{\text{the}}\;{\text{customer}}\;{\text{has}}\;{\text{Property}}\;{\text{of}}\;{\text{value - in - use}} $$

For instance, in the running business case, a transaction for the customer is a business travel. Hence, the ILS of the value-in-use could be as follows: “in *almost all* travels, the customer has *seamless* travel experience”.

##### Transaction perspective for a customer

By the term *transaction*, we understand the service instance that is being offered to the customer. For the TraXP business model, the transaction for the customer is a single *travel* that is being organized for him or her.

The transaction perspective for a customer allows to capture intended benefits, costs and their combination (return). These protoforms can be used to express expectations that desired benefits occur often, unacceptable costs are seldom, and overall return is positive:

4 $$ Q \left[ R \right]\;{\text{transactions}}\;{\text{have}}\;{\text{Property}}\;{\text{of}}\;{\text{benefit}} $$

5 $$ Q \left[ R \right]\;{\text{transactions}}\;{\text{have}}\;{\text{Property}}\;{\text{of}}\;{\text{costs}} $$

6 $$ Q \left[ R \right]\;{\text{transactions}}\;{\text{have}}\;{\text{Property}}\;{\text{of}}\;{\text{return}} $$

The following examples can be given for the running case:

- *Most* travels have *high comfort.*
- *A few complicated* travels have *major delays.*
- *Most* travels have *acceptable service/price ratio.*

#### Orchestrator-related protoforms

The orchestrator plays an essential role in a service-dominant business model. There is typically only a single orchestrator in a given instantiation of a service-dominant business model [66, 67]. The orchestrator typically aims at a high customer satisfaction due to the solution, and an achievement of benefits that outweigh the costs associated with each transaction, that is, high returns (financial or nonfinancial). To reflect these aims, we propose the following perspectives and corresponding protoforms.

##### Transaction perspective for an orchestrator

These protoforms are used to express expectations that desired benefits occur often, unacceptable costs are seldom, and overall profit is positive:

7 $$ Q \left[ R \right]\;{\text{transactions}}\;{\text{have}}\;{\text{Property}}\;{\text{of}}\;{\text{benefit}} $$

8 $$ Q \left[ R \right]\;{\text{transactions}}\;{\text{have}}\;{\text{Property}}\;{\text{of}}\;{\text{costs}} $$

9 $$ Q \left[ R \right]\;{\text{transactions}}\;{\text{have}}\;{\text{Property}}\;{\text{of}}\;{\text{return}} $$

The following examples from the business case are representative of this perspective:

- *Most* travels give an *acceptable kickback fee.*
- *A few very complicated* travels have *a very high document fees.*
- *Most* travels give *an acceptable return (profit).*

Please note that this is the same type of the protoform as in the customer case, but now the transactions of the orchestrator are being described.

##### Single-aggregated customer perspective:

The single-aggregated customer perspective allows to describe the benefits, costs and profits for all or certain groups of customers. Those statements are useful for the orchestrator to ensure that customers are satisfied with the services on a general, aggregated level.

10 $$ Q\;{\text{customers}}\;{\text{have}}\;{\text{Property}}\;{\text{of}}\;{\text{benefit}} $$

11 $$ Q\;{\text{customers}}\;{\text{have}}\;{\text{Property}}\;{\text{of}}\;{\text{costs}} $$

12 $$ Q\;{\text{customers}}\;{\text{have}}\;{\text{Property}}\;{\text{of}}\;{\text{return}} $$

Examples for the running business case include:

- *Most* of the customers can use *the service easily*.
- *A few* customers have *some additional fees.*
- *Most* customers have *good service/price ratio*.

Note that in the above protoforms, we do not use the qualifier *R* as all customers within one business model should be treated the same way.

##### Double-aggregated customer perspective

The double-aggregated perspective is a combination of the two perspectives: single-aggregated customer perspective and transaction perspective for customer. It allows for a global look for the orchestrator, yet with keeping the more individual view. These ILSs can be considered as meta-summaries of the transaction-perspective summaries.

13 $$ Q_{1} \;{\text{customers}}\;{\text{have}}\;Q_{2} \left[ {R_{2} } \right]\;{\text{transactions}}\;{\text{with}}\;{\text{Property}}\;{\text{of}}\;{\text{benefit}} $$

14 $$ Q_{1} \;{\text{customers}}\;{\text{have}}\;Q_{2} \left[ {R_{2} } \right]\;{\text{transactions}}\;{\text{with}}\;{\text{Property}}\;{\text{of}}\;{\text{costs}} $$

15 $$ Q_{1} \;{\text{customers}}\;{\text{have}}\;Q_{2} \left[ {R_{2} } \right]\;{\text{transactions}}\;{\text{with}}\;{\text{Property}}\;{\text{of}}\;{\text{return}} $$

The following exemplifies this perspective for the running business case:

- *Almost all* customers have *most* travels with *high comfort.*
- *Almost no* customers have *many complicated* travels with *major delays.*
- *Almost all* customers have *most* travels with *acceptable service/price ratio.*

#### Other party-related protoforms

The other parties (e.g., core and enriching) complement the business network and are essential to the functioning of the business model. To understand the performance or viability of a business model, considering the set of and balance between costs and benefits is typical.

##### Transaction perspective for a party

Similar to the case for the customer and orchestrator roles, the transaction perspective for a core or enriching party aims to capture expected benefits, costs and their combination (i.e., return or profit). An individual party can select objects, features that are most appropriate to express its strategic goals or motivations to participate. However, we should note that the transaction unit for each party might differ for the same business model instance. For instance, in the running case, while a transaction for the accommodation provider can be considered as “the stay of a traveler”, for the airline, this can be “the flight”, and for the insurance provider this can be “the insurance for the travel”.

16 $$ Q\left[ R \right]\;{\text{transactions}}\;{\text{have}}\;{\text{Property}}\;{\text{of}}\;{\text{benefit}} $$

17 $$ Q\left[ R \right]\;{\text{transactions}}\;{\text{have}}\;{\text{Property}}\;{\text{of}}\;{\text{costs}} $$

18 $$ Q \left[ R \right] transactions have Property of return $$

Based on the protoforms given above, the following examples can be given for an accommodation provider depicted in the running business case:

- *Most* stays have a *good price*.
- *A few* stays have *increased operational cost*.
- *Most* stays return *good profit.*

#### Network-related protoforms

KPIs can be generated also on the aggregated, network level, describing the benefits and costs of parties in the same or different roles in the business model.

##### Single-aggregated party perspective

To describe expected benefits, costs and returns (benefits with respect to costs) for a class of parties, we have the party perspective protoform. This protoform is similar to the single-aggregated customer perspective, but offers flexibility in terms of what parties can be selected. We can also describe the benefits and costs of all parties involved in the model.

19 $$ Q\;{\text{parties}}\left[ {{\text{of}}\;{\text{type}}\;X} \right]\;{\text{have}}\;{\text{Property}}\;{\text{of}}\;{\text{benefit}} $$

20 $$ Q\;{\text{parties}}\left[ {{\text{of}}\;{\text{type}}\;X} \right]\;{\text{have}}\;{\text{Property}}\;{\text{of}}\;{\text{costs}} $$

21 $$ Q\;{\text{parties}}\left[ {{\text{of}}\;{\text{type}}\;X} \right]{\text{have}}\;{\text{Property}}\;{\text{of}}\;{\text{return}} $$

The following KPIs are examples for the *insurers* in the running case:

- *Most* insurers have *additional customers.*
- *Almost no* insurers have *higher operational costs*.
- *Almost all* insurers have *acceptable profit*.
- *All* parties have a *decent profit (return).*

##### Double-aggregated party perspective

Similar for the double-aggregated customer perspective, the double-aggregated party perspective is a meta-summary of the transaction perspective and allows to group together parties with similar expected benefits, costs and returns.

22 $$ Q_{1} \;{\text{parties}}\left[ {{\text{of}}\;{\text{type}}\;X} \right]\;{\text{have}}\;Q_{2} \left[ {R_{2} } \right]\;{\text{transactions}}\;{\text{with}}\;{\text{Property}}\;{\text{of}}\;{\text{benefit }} $$

23 $$ Q_{1} \;{\text{parties}}\left[ {{\text{of}}\;{\text{type}}\;X} \right]\;{\text{have}}\;Q_{2} \left[ {R_{2} } \right]\;{\text{transactions}}\;{\text{with}}\;{\text{Property}}\;{\text{of}}\;{\text{costs}} $$

24 $$ Q_{1} \;{\text{parties}}\left[ {{\text{of}}\;{\text{type}}\;X} \right]\;{\text{have}}\;Q_{2} \left[ {R_{2} } \right]\;{\text{transactions}}\;{\text{with}}\;{\text{Property}}\;{\text{of}}\;{\text{return}} $$

Examples from the running case include:

- *Most* hotels have *many* stays with *good room prices*.
- *Almost no* hotels have *many last-minute* stay *cancelations of expensive rooms*.
- *Almost all* insurers have *good profit* on *almost all insurance contacts*.

### Use of the method to support decision making

**Fig. 8:**
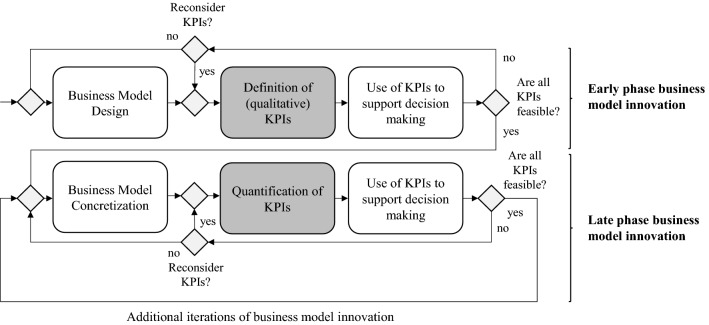
Application of method to support decision making in business model innovation

Figure 8 depicts how the proposed method can be used to support decision making for the business model innovation process. Accordingly, the following steps can be followed:

1. Each network actor (party) represented in the business model design should make explicit what strategic objectives or goals it desires to achieve with respect to the performance of the business model through participation in the business model design. These strategic objectives serve as the basis for the definition of KPIs.
2. Per strategic objective, each actor defines one or more ILSs (using the protoforms given above) that summarize or capture (part of) the essence of the respective strategic objective. Here, actors should take note of their respective role for the business model design (e.g., customer, orchestrator, party or reasoning from a network perspective) and leverage the respective set of protoforms accordingly. Through the use of the protoforms, the defined ILSs are catered to the business model design under consideration and are expressed in qualitative terms. The generated ILSs consequently serve as KPIs to support decision making. As a rule of thumb, to reduce the complexity for the decision-making process and to improve the practicality of the method, actors are encouraged to focus on the most important strategic objective and to define up to three (but at least one) soft-KPIs to operationalize this strategic objective. Accordingly, the generated set of KPIs (by all actors) remains manageable and digestible for further discussion and assessment of its feasibility.
3. The set of KPIs defined per actor is communicated across the business network and consequently serve as the basis for supporting the evaluation of the business model design. Accordingly, the business network discusses and judges for each KPI whether the KPI can realistically be satisfied or is deemed ‘rather feasible’. In case a KPI is not deemed feasible, either the KPI should be redefined (i.e., by selecting different quantifiers or summarizers if acceptable in light of strategic goals) or the business model design should be reconsidered.
4. As the business model concretizes, stakeholders can gradually quantify the KPIs to cater to the later phases of the business model innovation process, using the underlying membership functions for the linguistic variables. The gradually quantified KPIs are used to re-evaluate whether a business model design is acceptable, by assessing whether the quantified KPIs can be deemed ‘rather feasible’. Similarly, an infeasible KPI triggers a redefinition of the KPI or a reconsideration of the business model design. Step 4 is re-applied until the business model design is agreed-upon, i.e., the design is accepted and ready for implementation.

To provide a formal basis to our method and as a starting point for automated tooling, we have formalized the components used for our method (e.g., the SDBM/R and linguistic summarization) as well as their integrated use (to support techniques 1 and 2). We refer the interested reader to “Appendix” of this paper for the corresponding details of the formalization.

## First design cycle—initial application and evaluation of the method

In this section, we elaborate on the first design cycle of our method, which involved the application of the alpha version of the method to a business case drawn from practice to evaluate the validity of our method, and to generate initial results with respect to its utility. First, we describe the case study used and present the resulting service-dominant business model design. Next, we describe the application of our method, and how it is used to define business model-specific KPIs to support business model evaluation. Lastly, we discuss the evaluation setup and the initial feedback received for the alpha version.

### Description of the business case

The business case used for application and evaluation as part of the first design cycle originated from a mobility project in the Netherlands [23, 24] and concerns the traffic problems that occur in the city of Amsterdam when large public events are organized in the city. Amsterdam features several event locations that are closely grouped together. When large events (such as pop concerts or football matches) are held (often simultaneously and at peak hours, coinciding with everyday traffic), a significant inflow of traffic users is generated, which results in many severe traffic jams. As a consequence of these traffic jams, many negative externalities are generated (e.g., increased pollution, decreased business productivity and decreased city image). Given the severity of the traffic jams, the city sought after a collaborative solution aimed at mitigating the (effects of) traffic jams that emerge as a result of hosting large events. To do so, a business model workshop was orchestrated, involving many related parties such as the road authority, event providers, parking providers and retailers to explore how these parties may contribute or support a collaborative solution.

**Fig. 9:**
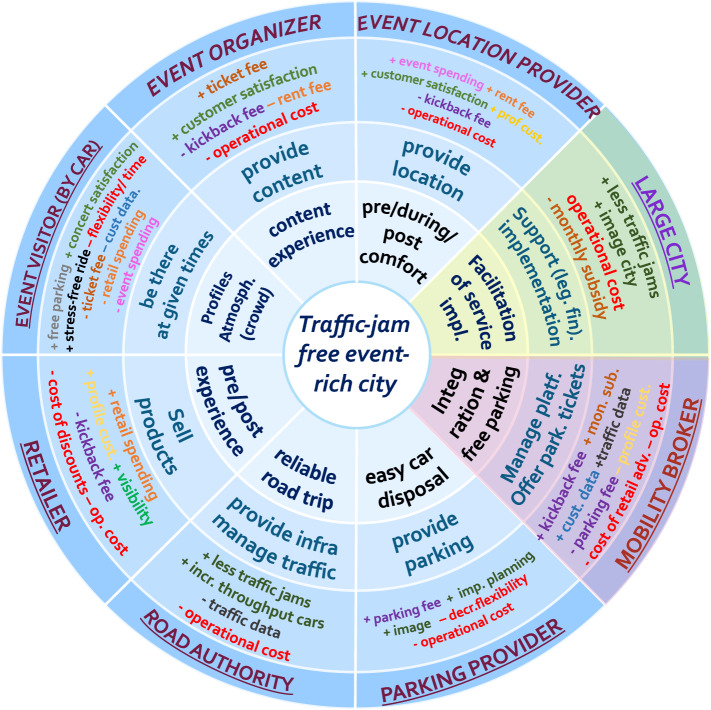
Business model design to address traffic jams due to large events

The resulting business model design is presented in Fig. 9 (modeled using the SDBM/R). The solution supported by this business model design constitutes of a service platform offered by a platform provider (referred to as *mobility broker*), which event visitors can access by means of their event ticket. Consequently, using their event ticket, event visitors can receive free parking tickets, which are valid at predetermined arrival times through this service platform. Accordingly, if event visitors arrive timely at the city and park their car at or before the predetermined arrival time, they will be eligible for free parking. Considering that parking is typically expensive in Amsterdam, such a scheme persuades event visitors to follow up on the predetermined arrival times and to arrive early at the city in order to enjoy free parking. As such, these predetermined arrival times can be set in such a way that the inflow of traffic is more balanced and the impact on traffic (depending on current and expected conditions) is decreased. This traffic information is forwarded by the *road authority* to the mobility broker to set the arrival times for free tickets accordingly.

To support the proposed solutions, the resources and services of several other parties are included, such as the *road authority* (to manage traffic information), the *parking provider* (to offer parking capacity), the *event visitor* (to use the service and arrive timely), the *event provider* (to offer the event) and the *event location provider* (to manage the location). To further stimulate the financial viability of the business model design, (associations of) *retailers* are involved as they may significantly benefit in terms of turnover from event visitors arriving early in the city if the business model design is to be implemented.

### Defining KPIs for the business case using the alpha version of the method

As the business model design is preliminary and in early phases of the innovation process, we apply our method to support the definition of *soft-*KPIs to support decision making and to clarify per actor under what conditions the business model design is strategically acceptable. Using the protoforms elaborated in Sect. 4.2.2–4.2.5, we define KPIs per actor in the business network. (Please note that not all protoform types are used, as some are not applicable for this business model.)

#### Customer-related KPIs (large city)

##### Value-in-use perspective

The first protoform for the customer (i.e., large city) concerns the value-in-use, which is *traffic-jam-free event rich city*. A feature of this value-in-use is the level of traffic jams, which can be characterized by three linguistic labels: heavy, medium and small. Hence, the following KPI can be defined based on this protoform:

- *On most* events, the *large city* has *few heavy* traffic-jams caused by the events.

##### Transaction perspective

The transaction perspective for a customer includes three types of summaries: describing benefits, costs, and returns per transaction or model execution. For the presented case, a transaction is considered as ‘orchestrating for a single event’. The benefit that can be quantified on the transaction (event) level is *less traffic jams*. Hence, the following example of an ILS is representative:

- *Most top-star* events have *few heavy traffic-jams caused by the events.*

In this business model, the large city pays a monthly subsidy irrespective of the number of events, so there are no costs for the customer per transaction. Similarly, it is not effective to define a return per transaction for the large city (i.e., the customer in the business model).

#### Orchestrator-related KPIs (mobility broker)

##### Transaction perspective

For every event, the orchestrator (mobility broker) is receiving kickback fees and data regarding traffic and visitors. The costs for the mobility broker pertain to payments in terms of parking fee. As no nonfinancial costs or benefits are specified, we can conclude that the orchestrator’s balance of costs and benefits is financially oriented.

Therefore, we can generate the following ILSs for benefits, costs and return:

- *Most* events generate a *significant* kickback fee from retailers.
- *Most* events generate customer data and traffic data of a *good* quality.
- *A few* events generate *very high* parking costs.
- *Almost all* events generate an *acceptable* profit (return).

##### Single-aggregated customer (large city) perspective

This perspective is relevant for the orchestrator particularly if the business model is to be implemented in a number large cities (with the same orchestrator). In this case, the orchestrator would be interested in monitoring their satisfaction with the following example statements:

- *Most large* cities have *more big* events.
- *Most large* cities have a *positive* image of the city.
- *Most large* cities have *less heavy* traffic jams.

##### Double-aggregated customer action perspective

The double-aggregated perspective helps us to generalize over different customers and repeated business model instantiations. In this business model, we have benefits expressed only on the transaction level. With the assumption that the orchestrator is involved in this business model in a number of cities, the following ILS are relevant:

- *Almost all* large cities have almost *no* heavy traffic-jams on *most of the* top-star events.

#### Other party-related KPIs (road authority, retailer, event provider, event location provider and event visitor)

The remainder of the parties (i.e., the core and enriching) are essential for the functioning of a business model. Hence, we make soft quantifications over the benefits, costs and returns for each of the remaining parties. For reasons of brevity, we present only some of the possible ILSs.

##### Transaction perspective for a party

For the *retailers*, it is important to have more customers and more purchases. We can generate ILSs for an individual retailer:

- *Most* big events generate *many* pre-event transactions.
- *A few* events have *high* costs of discounts.
- *Most* big events generate *good* profit.

#### Network-related KPIs

ILSs pertaining to the global perspective facilitate users to express the expected effect/profit of stakeholders that play a different role in the model but have the same objective or motivation. Such statements may be useful in communication to other parties, i.e., parties that can potentially participate in the business network.

##### Single-aggregated perspective

The single-aggregated perspective allows describing the benefits and costs for the group of stakeholders that play the same role in the business model. An example can be given for the retailers and event visitors:

- *Most* visitors have a *very high* event satisfaction.
- *A few* event visitors have *over-budget* event spending.
- *Most* retailers/parking providers make an *acceptable* profit.

We can also aggregate over parties playing a different role in the business model. A representative good example is as follows:

- *All* parties *participating for financial reasons (i.e., retailer, parking provider, event organizer, event location provider)* make a *good* profit.

##### Double-aggregated perspective

Below are a few examples of a mixed perspective type ILSs for (core) parties:

- *Most* parking providers have *significantly improved* planning on *most* events.
- *All* retailers make an *acceptable* profit on *most* events.
- *All* event organizers (location providers) have a *high* customer satisfaction on *most* events.
- *A few* retailers have a *small* number of transactions on *many small* events.

### Evaluation of the alpha version

To understand the perceived utility of the proposed method (e.g., what value the use of the method generates with respect to the problem it is designed for), we conducted three online workshops with groups of industry experts. In each workshop, at least three members of our research team were present to act as a moderator or facilitator.

To classify as an industry expert, we expected practitioners to have at least some degree of experience in business modeling, such that they would be able to determine critically or judge whether the method was deemed effective for its intended purpose (i.e., to support business model evaluation). As we focus on service-dominant, networked business models, we focused on inviting industry experts that either are active in service-oriented business domains, such as the mobility domain [66, 67], IT domain [49], or domains that are increasingly transitioning toward a service orientation, such as the agricultural domain [39]. In the end, we brought together 11 industry experts for our workshops (restricting each workshop to at a maximum four participants to support communication and discussion). The demographics for our workshops and participants are shown in Table 2.

**Table 2:** Demographics of industry experts for the set of workshops

Workshop	Expert	Tenure	Business modeling experience*	Domain
W1	Expert 1	More than 10 years	Knowledgeable	Consultancy (IT, Logistics)
	Expert 2	7–10 years	Very knowledgeable	Agriculture
	Expert 3	4–7 years	Somewhat knowledgeable	Consultancy
	Expert 4	Less than 2 years	Somewhat knowledgeable	Logistics
W2	Expert 5	4–7 years	Somewhat knowledgeable	IT
	Expert 6	More than 10 years	Somewhat knowledgeable	Consultancy (IT)
	Expert 7	More than 10 years	Very knowledgeable	Agriculture
W3	Expert 8	More than 10 years	Knowledgeable	Consultancy (IT)
	Expert 9	More than 10 years	Very knowledgeable	Consultancy (IT)
	Expert 10	4–7 years	Very knowledgeable	IT
	Expert 11	More than 10 years	Very knowledgeable	Consultancy (IT)

(*) The following scale was applied: not knowledgeable; somewhat knowledgeable; knowledgeable; and very knowledgeable

Each workshop took about 1.5 h to complete and was structured as follows. First, we explained the contents and working of our method, and how it is used to support business model evaluation. In case requested, we preluded our method description by an introduction on service-dominant business modeling, such that each industry expert had a sufficient understanding of the problem context. Consequently, we demonstrated the application of the method, for which we used the business case as described in Sect. 5.1, and presented the results as per Sect. 5.2. Any questions with respect to the demonstration or application of the method were resolved before eliciting the perceived utility of the method.

After the demonstration, we asked industry experts a number of structured questions with respect to the method’s utility. To concretize utility, we leveraged the theory on the Technology Acceptance Model (TAM), which is commonly used in the literature to understand the value of novel tools and technology [12]. Accordingly, we detailed utility through the constructs of *perceived usefulness* (value created by using the method), *perceived ease-of-use* (the degree that use of the method is free from physical or mental effort) and *intention-to-use* (the degree to which the user intends to use the method) [69]. Hence, in addition to the feedback received from discussions in the workshops, we asked industry experts to fill in a survey after the workshop, operationalizing TAM into four items related to *usefulness*, four items related to *ease-of-use* and two items related to *intention-to-use*. The full set of questions is presented in Table 3. Of the 11 industry experts that participated, 9 filled in the questionnaire.

**Table 3:** Set of questions used to assess utility of the proposed method

Evaluation construct	NR	Statement
Perceived usefulness	1	I think this method contributes to supporting the evaluation of service-dominant business models
	2	Use of soft-KPIs would enable me to better communicate my strategic preferences and goals**
	3	I do not see the added value of using this method*
	4	Overall, the I did not find the method useful to support the representation of strategic objectives as soft-KPIs *
Perceived ease of use	5	It would be easy for me to generate soft-KPIs using this method
	6	It was not clear to me how I should use the method to support the representation of strategic objectives or intentions as soft-KPIs*
	7	It would be difficult for me to apply this method*
	8	It was clear to me how this method should be used and what it is used for
Intention to use	9	I would use this method to support the representation of strategic objectives into business model-specific soft-KPIs or qualitative statements
	10	I would not use this method in favor of already known methods to generate business model KPIs

*Questions indicated with a star (*) are deliberately inversed

**Note that for simplicity and ease of understanding, we labelled the ILSs as ‘soft-KPIs’ that capture the strategic intent or desires of a respective stakeholder

### Results of the alpha evaluation

The results of the surveys are presented in Table 4. As shown in the table, the method is generally positively received. In the subsequent sections, we discuss the results per utility criterion in detail. This discussion serves as the input for improving our method further.

**Table 4:** Results of surveys with respect to the utility of proposed method

Criteria	Question	Strongly disagree	Disagree	Neutral	Agree	Strongly agree
Perceived usefulness	1	0	0	0	9	2
	2	0	0	1	9	1
	3*	0	0	1	5	5
	4*	0	0	1	8	2
Perceived ease of use	5	0	0	6	5	0
	6*	0	1	0	8	2
	7*	0	0	5	6	0
	8	0	0	0	10	1
Intention to use	9	0	0	3	7	1
	10*	0	0	1	8	2

(*) Responses are reversed to account for the negative form of the question

#### Perceived usefulness of the alpha version of the method

With respect to perceived usefulness, most industry experts found our method to be useful to support decision making for business model evaluation. In particular, industry experts are appreciative of the soft or more qualitative nature for defining KPIs:

*“It indeed really does not make sense if you talk about new business models to discuss the details in a quantitative way already. I experience the same at our company. We have targets like 5% year on year savings or something like that for a given business time, you cannot quantify it from a given business case already years ahead, it does not make any sense. You can set the target, but you cannot show that you realize it, it is not very easy.” [Expert 5]*

However, some experts indicated that, as a result of the more subjective nature for the KPIs (depending on the interpretation of a respective stakeholders), collaboratively judging the feasibility of the KPIs could be difficult and could become subject to misinterpretations. This requires ample support in terms of how such linguistic variables are defined (with respect to their membership function), and how these later on are to be quantified.

*“Making the KPIs objective in use is of course more difficult and could potentially lead to misinterpretations and unnecessary discussions (for one stakeholder it may be summarized as ‘a lot’, whereas for others this may imply ‘some’).” [Expert 4].*

#### Perceived ease-of-use of the alpha version of the method

With respect to perceived ease-of-use, the results are generally positive, although in contrast to perceived usefulness they are more skewed toward a *neutral* perspective. Industry experts indicated that the method was intuitive and could be comfortably applied for their own business initiatives, although some experts indicated that further practice would be needed, hinting at additional support or example business case applications.

*“I would have to work with this technique—a set of exercises would be good to get a better feeling for the technique.” [Expert 1]*

*“Consider generating some best practices to make it even easier for users to use the technique.” [Expert 5]*

#### Perceived intention-to-use of the alpha version of the method

With respect to intention-to-use, we see that the survey results are generally positive. The benefits offered by the method (e.g., supporting decision making through KPIs that can be catered to the characteristics of the innovation process) are generally appreciated.

*“I think it is definitely worth it to try out this technique in the future.” [Expert 2]*

## Second design cycle—hands-on application and evaluation of the method

Based on the feedback received in the first round of evaluation, we have improved our method (from alpha to *beta version*, as presented in this work). In the second evaluation round, the method has been applied in a real-life business case, in which two project managers pertaining to the case applied the method to define and quantify KPIs for the general stakeholder roles in a specific business model design. In the following, we first describe the business case used for the hands-on application of the method. Next, we describe the results of the application, indicate the soft-KPIs that were defined, and discuss the results with regard to the utility evaluation of the beta version.

### Description of the second business case used for the beta evaluation

The second business case originated from a European-funded project in the horticulture domain. The project focused on offering support to both small and large growers to improve their greenhouse production, through digitally enabled services (i.e., without the grower having to purchase or acquire hardware) aimed at improving decision making in such production processes. Such services leverage techniques such as machine learning or artificial intelligence (AI) to help growers better understand their production process and as a result to make better decisions with regard to producing and managing their products (e.g., plants, fruits). As such services entail complete solutions for the customer, integrating the resources of stakeholders, such as the AI tech providers, sensor tech providers and consultancy providers, the project team explored the structure and feasibility of such initiatives through service-dominant business modeling. Accordingly, two business model workshops were organized to design business model blueprints that would describe such collaborations.

**Fig. 10:**
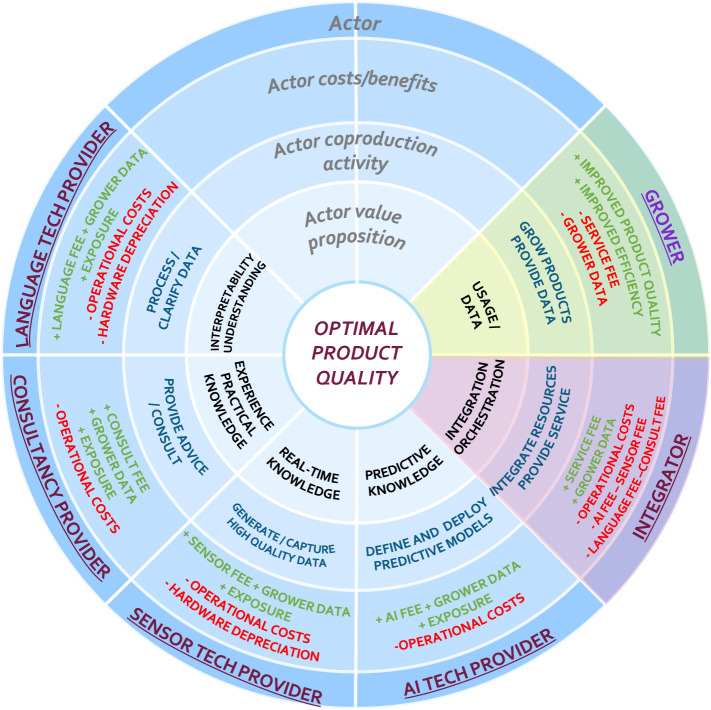
Service-dominant business model design for 'optimal product quality'

The resulting business model design is presented in Fig. 10. The *grower* is considered as the *customer* for the business model, whereas *the integrator* takes the role of the orchestrator. The integrator is responsible for orchestrating the network and integrating the services of the parties in the business network into a holistic solution. The solution aims at improving the product quality of the grower (through better, informed decision making). The grower contributes to the central value-in-use by providing usage data, such that the service can be steered and optimized. The remainder of the network is composed of an *AI tech provider* (contributing value in terms of predictive knowledge gained with the use of machine learning or AI models), a *sensor tech provider* (adding value in terms of real-time knowledge through sensor application in the production process of the customer), a *consultancy provider* (contributing value in terms of practical knowledge and experience on growing processes) and a *language tech provider* (adding value through improving the understandability and interpretability of generated data and outcomes).

### Evaluation setup

After the business model design workshop, a second workshop was organized with the participation of two practitioners, who served as the project managers for the solution development (in addition to serving as the representatives of the *Integrator* and *Language/AI tech provider* in the first workshop). The objective of the workshop is to clarify the strategic intent of the stakeholders at the preliminary phase of the business model innovation, by applying the beta version of the method for defining KPIs.

Given their leading role in the project, both participants have ample understanding of the motivations and perceptions of each stakeholder present in the business model blueprint. In addition, both project managers have considerable knowledge on business modeling and development, in turn are able to translate strategic motivations into more concrete objectives. The workshop was moderated by two researchers from the author team, providing guidance and steering on the method application where needed.

The workshop was structured as follows: First, both stakeholders were introduced with the working of the method (i.e., the summarization of quantitative intentions into qualitative terms, use of the protoform catalogue, and the subsequent quantification of KPIs). Next, using the business model blueprint as a basis (Fig. 10), both stakeholders were encouraged to use the method to define KPIs that would capture or make explicit their strategic intent. Here, the protoform catalogue was reintroduced such that stakeholders could draw on templates if so desired.

The workshop was concluded with a semistructured interview on the usefulness and ease-of-use of the method, eliciting the pros and cons of method use. We also inquired whether stakeholders would intent to use the method in future business model endeavors. We built on the interview guidelines provided by Rowley [54] to structure our interview. The hands-on workshop took roughly 1.5 h to complete and was orchestrated online (as a result of the COVID-19 pandemic). Afterward, the semistructured interview took about 45 min to complete. Both the workshop and interview were recorded. We used content analysis [36] to structure the analysis of the results.

### KPIs defined for the second business case

Using the method, the participants (two project managers) jointly defined KPIs per each role in the business model blueprint (Fig. 10), which made explicit the conditions under which a stakeholder would be willing to participate in the business model. As the business model blueprint was preliminary in nature, the KPIs are represented in a qualitative way, summarizing quantitative intentions into qualitative statements (ILSs). In the following, we describe per generic role these soft-KPIs (or ILSs).

#### Customer-related KPIs (grower)

To increase the competitive position of the *growers* (customer), the importance of increased efficiency in production processes (related to the accrued benefits for the grower) as well as increased quality of the resulting products (related to the value-in-use) were stressed. In addition, the importance of generating knowledge on the plants (to improve decision making) was emphasized in discussion. Accordingly, these items were selected as the objects for the definition of soft-KPIs. In addition, the *transaction* or *service* was considered as the *growing cycle/production cycle* (production process iteration for a set of plants or fruits) involving the use of the service (*service invocation*).

Using the protoforms (3) and (4), the following KPIs were defined:

- *Most service invocations* generate *high quality information with respect to the plants.*
- For *most service invocations*, *almost all growers* generate *moderate time savings.*
- *Most service invocations* result in *increased flexibility for the customer.*
- *Most of the plants* should have *an acceptable height.*

#### Orchestrator-related KPIs (integrator)

Focusing on the customer perspective, the project manager representative for the integrator deemed that the service, in addition to improving the quality of products for the customer, should also be reliable and efficient for the customer to use. Accordingly, using (13), the following KPI was defined:

- For *many* of the service invocations, *many* customers experience *limited complications* using the service.

In addition to the customer perspective, the integrator considered that profit generation and acquisition of customer and sensor data (to use in different business models) were deemed important for participation. Using the service invocation as a transaction, the following KPIs (leveraging the protoform 10) were defined to address this:

- *Most service invocations* generate an *acceptable monthly profit.*
- *Almost every* service invocation generates *significant amounts of customer data/high amounts of sensor data.*

#### Other party-related KPIs

For the AI Tech Provider, Language Tech Provider and Consultancy Provider, the importance of generating high-quality information due to participating in the business model was stressed. Such information (generated through the grower using the service) would help them in return to improve their own service propositions (for instance the development of prediction or interpretation models) and as a result be beneficial in future applications for different business cases. As a result, the following KPIs, building on (16), were defined for these parties:

- *Most service invocations* generate *high-quality information/knowledge.*
  In addition, parties indicated that the generation of profit was important to sustain participation in the business model design. Accordingly, using (16), the following KPI was defined:
- *Many service invocations* result in *significant return.*

#### Network-related KPIs

Lastly, the stakeholders considered that the business model design should generate significant exposure in terms of positive awareness, which—in turn—would improve the image of the stakeholders involved. Accordingly, the following KPIs were generated, using (19) as a basis:

- *All parties* generate *increased (company) image* by participating in the business model.
- *All parties* generate *high learning* through participation.

#### Feasibility of KPIs and quantification

As per the last step of the method, the participants assessed whether the generated KPIs could be deemed ‘feasible’, implying that the business model design in its current form is acceptable for all stakeholders and thus may serve as the basis for further concretization. Given the expected benefits of the proposed service (increased product quality and increased efficiency through time savings and improved decision making) and the expected generation of data through participation, the participants considered KPIs to be feasible. Here, they also highlighted the importance of addressing the preliminary quantification of costs and benefits as a next step, as some of the KPIs were explicitly profit-oriented. Whilst it was expected that parties would generate acceptable returns through participating in the business model, such claims should be supported through data to truly verify this.

As the business model design was still preliminary (i.e., the stakeholders had limited insights on the further quantification of the design), at this stage the quantification of KPIs was explored as an exercise for future concretization, rather than a more concrete input for decision making. Nevertheless, the stakeholders focused on how linguistic variables set for KPIs could be further quantified. Considering the KPI ‘for *most service invocations*, *almost all growers* generate *moderate time savings*’, the integrator worked on quantifying the time savings for the grower and what *moderate savings* would refer to in light of the customer. Ultimately the integrator concluded that *moderate time* savings would constitute savings of *about 30 min* per day. Here, a range between 25 and 35 min could be considered to account for flexibility. Such savings could considerably help the grower to improve efficiency for the production process, but would not be considered as overly high or low (as this would correspond to savings to roughly 60 min or less than 10 min, respectively).

### Utility evaluation of the beta version of the method

After the completion of the second workshop, we conducted a semistructured interview with two workshop participants. Similar to the case for the alpha evaluation, we used the operationalized constructs of TAM (*perceived usefulness, perceived ease-of-use* and *perceived intention-to-use*) as the basis when asking questions. In the following, we describe the results of this interview, providing detailed feedback on the components of the method (i.e., the definition of soft-KPIs, catalogue of protoforms and the quantification and verification) where applicable. We use quotes to support the findings.

#### Perceived usefulness of the beta version of the method

With respect to the usefulness of the method, both stakeholders agree that the hands-on application was considered valuable and helped to address an important challenge for the development of any novel business initiative, as it stimulates or even directs actors to be explicit about how they define success for the business model:

*“I can see this for our project as well. Some of the partners are quite technically-oriented, for which thinking in business development is far away from their traditional thinking. Talking to the partners of the project and asking what their function, relationships or benefits are for the project,… sometimes they do not even really know because they do not think about it. Focusing on such KPIs can help for this …, clearing up what direction to develop, realizing what values and goals for each partner are, and stimulating partners to think about it …”. [Expert 13]*

Zooming-in on the soft-quantification of KPIs, both stakeholders appreciated the fact that the KPIs are initially defined qualitatively in this early phase, as this would give stakeholders more time to think about what directions to take:

*“It helps in getting the right feeling for the direction, if you make it qualitative early on and then slowly work towards quantitative values it can help you think about it a bit more, and as result help you to push you in the right direction” [Expert 12]*

In addition, stakeholders indicated that it may help actors to better express their desires or preferences given the use of natural language for such KPIs:

*“I think it is very helpful. It is a little bit more abstract, therefore not so concrete, and I believe that actually helps the people involved to better express their opinion in such settings”. [Expert 13]*

Positing whether such qualitative expressions could also make it difficult to interpret things or that it may result in less objective statements, stakeholders indicated that although this can be the case, this is understandable for early design phases.

*“I would say that this [difficulty of interpretation and loss of objectivity] is standard and understandable. Especially in such early phases, you have to get in the right direction and make some decisions. This is the only way to get more into this development and thinking process. I think you need to describe this, making it qualitative where needed to support this and to quantify this as you go. You need people to generate a description of what value to propose and what goals to achieve.” [Expert 12].*

#### Perceived ease-of-use of the beta version of the method

With regard to the ease-of-use of the method, participants indicated that the method was in general clear and that the steps help in structuring the application of the method. However, they also indicated that it would take some time to become skillful in the method use, as it was considered comprehensive. Accordingly, stakeholders indicated that it would be helpful to generate even smaller blocks of activities, complemented by examples and summaries, to further improve the use of the method:

*“For me, the method application was more or less clear. However, I think the method can further benefit from small blocks or steps and examples on the way. The method is large and presents a lot of information. In the current set-up, you get to the details somewhat quickly and this may make it difficult to think about it and model it. Adding more breaks in between and given examples can help, also for people less common in business development, to make it easier to use.” [Expert 12].*

Focusing on the catalogue of protoforms as a means to support the definition of KPIs, both stakeholders agreed that the catalogue is helpful, in the sense that the catalogue serves as a starting point and can support clarifying, particularly for inexperienced users, what kind of KPIs can be expected:

*“I feel these templates are helpful for all parties, even those that may have less experience in business development. Using the templates, parties can already think about it in more detail, can obtain a better understanding of their role for the system and position KPIs accordingly.” [Expert 13]*

With regard to how easy it was perceived to quantify KPIs, both stakeholders deemed it more or less clear. Again, examples and additional practice would be beneficial here:

*“More or less clear, but I have to practice it.” Additional examples and practice would help in further supporting this.” [Expert 13]*

#### Intention-to-use of the beta version of the method

With regard to the intention-to-use, both participants were positive. As the business case was rather preliminary, they indicated that it would be valuable to conduct follow-up applications of the method, actively involving represented actors for the business network, to make explicit what drivers are present for each of the actors.

*“I think it would be valuable to do another workshop with this method, where we can refresh the contents and results generated with other parties and to think about it again together. I think this is very important for our system, that we make explicit what benefits each partner desires to obtain, to define these to make progress for our project.” [Expert 13]*

*“We will gradually be better able to see how the collaboration will work and what the role for each partner will be. As a result, it also becomes important to make things explicit; for example, understanding what customers are willing to pay for the service and why they are willing to pay for this. I think using this method can help us to take that next step and go to the next step of business development.” [Expert 12].*

## Conclusions

In this paper, we have focused on the development and evaluation of a method supporting the definition of business model KPIs that can be catered to the characteristics and requirements of the business model innovation process. Previous research has demonstrated the importance and value of defining KPIs in business model evaluation to support decision making. Such a practice helps users to make strategic goals or intentions explicit and offers structure with respect to interpreting the expected performance of business models. However, we observe that available techniques supporting the definition of KPIs tend to focus on quantitative KPIs, which offers limited support for decision making in early phases of business model innovation. Our method addresses these concerns by offering support for the definition of *soft*-KPIs suitable for early-phase decision making and clarifying how these KPIs can be gradually quantified to support decision making in later phases of the innovation process.

In this work, we describe how *soft*-KPIs—in the form of *intentional linguistic summaries*—can be generated on the basis of a business model design. To offer further guidance, we have complemented this by providing a catalogue of standardized templates catered to various stakeholder roles in service-dominant business models. Accordingly, depending on the generic roles that a user may represent, such templates can serve as a starting point for the definition of KPIs.

Lastly, we describe how the soft-KPIs can be further quantified to provide support for decision making throughout the business model innovation process. To assess the utility of our method, we have evaluated it through a set of two real-life business cases, involving the opinions and feedback of 13 industry experts. The results indicate that the method is considered useful in the context of business model decision making and that practitioners appreciate the ability to represent quantitative intentions into more qualitative yet structured statements.

Our research has important contributions both for research and for practice. For research, our method contributes to the ongoing call for supporting decision making in business model innovation [15, 61]. Whilst research on business model innovation has increased significantly over the past decade, clarifying its relative structure and antecedents, supporting decision making in business model innovation is still under-investigated [55]. Our work contributes to closing this gap, offering holistic support toward decision making in business model innovation, bridging the gap between purely qualitative and quantitative decision making. In turn, the method can be used to explore and better understand intermediate phases of business model innovation, which can partially be supported through data but are still highly uncertain with respect to their outcomes. In addition, our method contributes to the existing work on the definition of KPIs [26, 52], offering a novel structured approach for the generation of qualitative KPIs and their subsequent quantification.

Our research has important implications also for practice. Practitioners that are involved in business models design and innovation will benefit from the support that our method offers in the generation of soft-KPIs for enhanced decision making. This is particularly relevant for early-phase decision making, which often lacks formal, structured support. Hence, even though the business model design is at an early phase and possesses high uncertainties, the quantitative intentions or perceptions can still be taken into account without the need to explicitly start quantifying the expected business model performance. More so, the resulting KPIs can be gradually quantified as the business model is implemented and put in operation, allowing them to be used as the basis for monitoring the performance of implemented business models.

Our research also has some limitations. First and foremost, our method takes the SDBM/R technique at its foundation for the definition of KPIs for service-dominant networked business models featuring multiple partners. This is reflected on the specific role perspectives that are introduced and the way the KPIs can be defined for each. Although the method can be extended or adapted to accommodate other business model design tools (such as the BMC or SBMC), in its current form it might pose challenges for its potential users that are inexperienced with the SDBM/R technique. In other research [65–67], we have observed positive feedback with respect to the use of the SDBM/R technique, which mitigates this limitation. Nevertheless, future research can dedicate its efforts to adapt the method for different business model design approaches.

Second, with regard to the evaluation of the beta version of the method, not all stakeholder roles were represented in the set of workshop participants, which influenced decision making on the feasibility of the resulting KPIs. Whilst the application of the method demonstrated that stakeholders were able to define KPIs catered to their needs, in general all stakeholders should be present to truly determine whether the business model design is acceptable. In addition, even though the evaluation of the beta version of the method facilitated stakeholders to gradually quantify KPIs (providing evidence that the method can be used in later phases), the business model design under consideration was still preliminary in nature, making it difficult to explicitly determine how KPIs should evolve or be quantified. To further strengthen and refine our method, additional case studies, particularly those that cover the use of KPIs for the entire business model innovation process, should be conducted, providing further evidence for the utility of the method in later phases of the innovation process.

Furthermore, as also highlighted by the results of our evaluation, we predominantly focus our intentional linguistic summaries on the viability (e.g., costs and benefits) of a business model design. Although the business model viability is often considered as the most important driver of any novel initiative [46], other factors, such as the feasibility or robustness of a business model design, should not be neglected. Therefore, future research should also dedicate its efforts toward supporting the evaluation of the feasibility of business models in the light of our proposed method.

Future research can also investigate how our method can be adapted for the definition of KPIs for *generic* or *reference type business models* [18], which describe a generic business logic but without a concrete information about the stakeholders that fulfill such roles. For the presented method, we consider the set of stakeholders participating in the business model to be largely concrete, allowing individual parties to use the method to define KPIs that suit their strategic objectives. However, for such generic cases or reference models, the method cannot built on the input of such concrete stakeholders. Still, generic performance indicators are desired to understand the conditions under which such reference business models are valid or viable, such that such reference models can better support decision making or help in exploring new business models. To do so, research can explore how the presented method can be adapted or how the method can be used to accommodate this.

## Appendix: Formalization of the proposed method

To support the systematic application of our method, and to serve as the basis for the development of automated tooling in future research, in this section we formalize our method. Our formalization is built upon the two main elements of this technique: business model radars modeled using the SDBM/R technique, and intentional linguistic summaries. Accordingly, we first formalize the SDBM/R concept and then we formalize the ILS concept. Consequently, to provide a formal backbone to our method, we integrate both formalizations to support our method. We have presented the initial version of this formalization in Gilsing et al. [19–21]. In this section, we presented the extending formalization, accommodating the different protoforms presented for the catalogue (as per Technique-2).

### Formalizing the SDBM/R

In this section, we formalize the graphical business model representation SDBM/R, as shown in Fig. 1. We do this by defining a formal conceptual data structure labeled SDBMR. SDBMR has an overall structure that is independent from the number of involved parties in the model. Each party shares the same structure. This overall structure is also reflected in the formalization, which is provided in two steps: the formalization of the business model radar structure and the formalization of a party as an element in the radar structure.

The business model radar type (SDBMR) is a business model specification tuple with the following formal type and constraint:

25 $$ SDBMR = \left\langle {name:L,\;value:ViU,\;cust:P,orch:P,parts:\left\{ {\langle part:P,core:BOOL\rangle } \right\}} \right\rangle $$

26 $$ parts \ne \emptyset $$

In this formalization *name* is the name of the business model from the set of *labels L*, *value* is the value-in-use of the business model from the set of *values-in-use ViU*, *cust* is the customer from the set of *parties P*, *orch* is the orchestrator party from *P*, and *parts* is the set of other parties of type {〈*P,BOOL*〉}, i.e., a set of tuples each containing a party and an indication whether the party is a core party in the business model. This formalization structure states that exactly one customer party is present and exactly one orchestrator party. The constraint (Eq. (27)) specifies that at least one other party is present making it a true networked business model and not a dyadic relation.

A SDBMR instance b therefore has the following format:

27 $$ b = \langle l,viu,p_{1} ,p_{2} ,\left\{ {\langle p_{3} ,b_{3} \rangle , \ldots ,\langle p_{n} ,b_{n} \rangle } \right\}\rangle , n \ge 3 $$

A party is the specification of a role in a business model radar with the following type:

28 $$ P = \left\langle {name:L,avalp:\left\{ {AVP} \right\},acopa:\left\{ {ACA} \right\},aben:\left\{ {AB} \right\},acost:\left\{ {AC} \right\}} \right\rangle $$

29 $$ avalp,acopa,aben,acost \ne \emptyset $$

Here *name* is the name of the party from the set of *labels L*. *avalp* is the set of *actor value propositions* of a party (a party can have more than one actor value proposition), *acopa* the set of *actor coproduction activities* (a party can have more than one activity), *aben* the set of *actor benefits*, and *acost* the set of *actor costs*. The additional constraint [Eq. (30)] specifies that all of the four sets need to be non-empty for a business model to be viable: each actor needs to contribute to the central value-in-use, each actor needs to perform at least one activity to generate this contribution, and each actor needs to have both benefits (its reason to participate in the business model) and costs (not to be a ‘free rider’ to the other parties).

This formalization already provides an initial set of simple, automatically checkable correctness criteria for business models specified using the SDBM/R technique. It should be noted that these criteria are of a syntactical nature and do not specify the intended business effects of the business model. This can be achieved with intentional linguistic summaries (ILSs).

### Formalizing linguistic summarization

Intentional linguistic summaries share the same structure with linguistic summaries. They are qualitative statements or propositions, summarizing quantitative intentions, together with a quality value. First, we present the overall structure of linguistic summaries. Next, we discuss their components in detail.

The set of linguistic summaries LS has the following type:

30 $$ LS = \left\langle {qs:QS,qual:\left\{ {\left\langle {qname:L,qval:\left\{ {REAL,L} \right\}} \right\rangle } \right\}} \right\rangle $$

Here *qs* is *quantified statement* of type *QS*, *qual* is set of *quality values* of type {〈*L*, {*REAL*, *L*}〉}, i.e., a set of pairs consisting of a label of a quality measure and its value (a number or a label, e.g., “feasible” in case of expert evaluation).

The quantified statement *QS* has the following type:

31 $$ QS = \left\langle {quant:QF,obj:\left\{ {OB} \right\},oqual:\left\{ {OQ} \right\},ochar:\left\{ {OC} \right\}} \right\rangle $$

Here *quant* is a *soft quantifier* of type *QF*, *obj* is the set of *quantified objects* of type *OB*, *oqual* is the set of *object qualifications* (features) of type *OQ* (known as qualifiers), and *ochar* is the set of *object characteristics* (features) of type *OC* (known as summarizers). Object qualification *oqual* can be a feature describing all objects in a UoD. Please note that in our context an Intentional Linguistic Summary is of type *QS*.

Therefore, *qs*, an instance of quantified statement *QS* has the following format:

32 $$ qs = \langle qf,ob,oq,oc\rangle $$

Now we discuss the components of the quantified statements.

The first component, *QF*, is the set of soft quantifiers. The quantifiers describe the quantity that the objects have a certain property. Usually relational quantifiers are used in linguistic summarization (i.e., describing the proportion within the set). *Most* or *above 70%* are examples of relational quantifiers. Absolute quantifiers (i.e., referring to an absolute object count), such as *around 5*, *more than 10*, are seldom used.

In many papers, the following set of quantifiers is used [14, 30, 73]:

$$ QF_{ou} = \left\{ {ALL,\;ALMOST\;ALL,\;MANY,\;SOME,\;FEW,\;ALMOST\;NONE,\;NONE} \right\} $$

We use this set in the intentional linguistic summaries for our method.

The next component is the set of *quantified objects OB*. The type of *OB* is the powerset of objects in the UoD (so the set of possible sets of objects) over which we want to state the quantified statements:

33 $$ OB = \left\{ {\left\{ {O \in UoD} \right\}} \right\} $$

Consequently, a set of quantified objects ob is a set of elements in the UoD:

$$ ob = \{ o \in UoD\} $$

A feature of an object is a tuple of type *F* that contains the feature label and the set of linguistic value labels:

34 $$ F = \left\langle {featureLabel:FL,\left\{ {linguisticValue:LV} \right\}} \right\rangle $$

For instance, a feature *color* can be formally defined as

$$ f = \left\langle {``color'',\left\{ {``red'',\;``green'',\;``blue'',\;``black'',\;``white'',\;``yellow'' } \right\}} \right\rangle $$

Linguistic value labels can be defined and represented as fuzzy sets, where *M* is the membership function of this fuzzy set. Membership function quantifies a degree to which an element belongs to a set.

35 $$ M:OB \times FL \times LV \to \left[ {0,1} \right] $$

The membership functions do not have to be defined for intentional soft quantified statements at the early design stage of business models (ideation), allowing the linguistic value labels to have more intuitive definition and meaning and be made more precise in later design stages.

The set of features of an object is given by the function *ofeat* that takes an object:

36 $$ ofeat:UoD \to \left\{ F \right\} $$

Every feature is associated with an enumerated set of possible linguistic values. In principle, a feature can possibly have multiple linguistic values with different membership values – but we abstract from this construct. For example, we take object *c ∈ Cars*:

$$ ofeat\left( c \right) = \left\{ {\langle ``color'',\;``red''\rangle ,\langle ``speed'',\;``fast''\rangle ,\langle ``class'',\;``luxury''\rangle } \right\} $$

The set of object qualifications *OQ* consists of pairs of a feature label and a linguistic value. In general, more complex situations are allowed, where multiple feature labels and linguistic values can be combined with conjunctions. For pragmatic reasons, in this work we focus only on the simple case.

37 $$ OQ = \langle featureLabel:FL,linguisticValue:LV\rangle $$

A function *oqmem* identifies subsets from the UoD that have a certain property, characterized by a linguistic value of a feature:

38 $$ oqmem:OB \times FL \times LV \to OB $$

Hence, an object qualification *oq* is applied to a set of objects to constrain this set to a subset under consideration.

The object characteristics *OC* is the last element in the quantified statement structure. *OC* contains pairs of a feature label and a linguistic value, similar to *OQ*. In general case, more complex expressions of feature labels and linguistics values are possible, but again for reasons of simplicity and pragmatism, this is beyond the scope of the current formalization.

39 $$ OC = \langle featureLabel:FL,linguisticValue:LV\rangle $$

In short, *OC* is an intentional predicate (characteristics) over *QF* (quantity) objects resulting from *oqmem*, i.e., subset of UoD that have a certain property expressed by *OQ*.

The above formalism describes the structure of linguistic summaries and quantified statements suitable for an automated tool. However, statements in this form are not easily comprehensible or interpretable. For the users, we can generate the linguistic summaries in a textual representation, hence using a natural language. For instance, a quantified statement:

$$ qs_{1} = \langle MANY,Cars,\langle ``color'',``red''\rangle ,\langle ``speed'',``fast''\rangle \rangle $$

can be represented as “*MANY red* cars are *fast*”. An example of a quantified statement in a simplified from can be

$$ qs_{2} = \langle SOME,Cars,\langle any\;Feature,\;all\;Values\rangle ,\langle speed,fast\rangle \rangle $$

and can be represented textually as “*SOME* cars are *fast*”. In this case, $\langle any\;Feature,all\;Values\rangle$ is a feature describing all objects in a UoD.

### Formalization of the catalogue of protoforms (technique 2)

In this section, we discuss how we combine the formalisms of business model radars and intentional linguistic summaries into an integrated formalism for the specification of intentional linguistic summaries for business models, to support the evaluation of business models.

To generate intentional linguistic summaries for specifying intentions of business models, we propose a set of templates that represent typical characteristics of business models, which follow the *QS* structure as introduced above.

Given a SDBMR instance *b* (as introduced in the Sect. 4.2.1):

$b = \langle l,viu,p_{1} ,p_{2} ,\left\{ {\langle p_{3} ,b_{3} \rangle , \ldots ,\langle p_{n} ,b_{n} \rangle } \right\}\rangle$ we want to specify *QS* instances over this SDBMR instance that represent ILSs and thereby create a soft-quantified *SDBMR* with the following type (merging the formal specifications of *SDBMR* and *QS* shown in previous sections):

40 $$ SQBMR = \langle bmr:SDBMR,sq:\left\{ {QS} \right\}\rangle $$

An instance *s* of the type *SQBMR* is a soft-quantified business model radar.

$$ s = \langle b,sq\rangle $$

It is a pair of a business model *b* and a set *sq* of quantified statements, $sq = \left\{ {qs_{0} ,qs_{1} , \ldots ,qs_{p} } \right\}$, describing the desired soft-quantified behavior of *b* when it will be executed in practice. This formalization allows the precise specification of the nature of these ILSs to obtain a structured soft-quantification and to reason about the set of ILSs. To do so, the ILSs are organized in categories with respect to the customer, the orchestrator, and the remainder of the parties (e.g., *core* and *enriching*) as we have described above. Below, we map the informal structures of Sects. 4.2.2–4.2.5 to formalized structures using the formalism shown in this section and introduced in Gilsing et al. [72].

#### Customer-related protoforms

There are two basic types of customer-related ILSs, described in Sect. 4.2.2.

Below we show the formalization of those ILSs:

*Value-in-*use* perspective:*

The protoform “In *Q* transactions the customer has *property of value-in-use*” is formalized as:

41 $$ qs_{0} = \left\langle {qf_{o} ,p_{1} \left( {transaction} \right),\langle any\;Feature,all\;Values\rangle ,f\left( {viu} \right)} \right\rangle $$

with $$qf_{0} \in \left\{ {ALL,ALMOST\;ALL, MOST} \right\}$$.

The example “*almost all* travels, the customer has *seamless* travel experience” hence is represented as *qs*_*0*_ = 〈*ALMOST ALL*, customer (travel), 〈*any Feature, all Values*〉, “*seamless* travel experience”〉.

*Transaction perspective for a customer:*

42 $$ qs_{1a} = \langle qf_{1a} ,transaction,f\left( {transaction} \right),f\left( {p_{1} .aben} \right)\rangle $$

43 $$ qs_{1b} = \langle qf_{1b} ,transaction,f\left( {transaction} \right),f\left( {p_{1} .acost} \right)\rangle $$

44 $$ qs_{1c} = \langle qf_{1c} ,transaction,f\left( {transaction} \right),f\left( {p_{1} .aben,p_{1} .acost} \right)\rangle $$

with $$qf_{1a} ,qf_{1c} \in \left\{ {ALL,ALMOST\;ALL, MOST} \right\} $$ and

$$ qf_{1b} \in \left\{ {NONE,ALMOST\;NONE,FEW} \right\} $$

By using two different sets of quantifiers, we want to emphasize that ILSs should form summaries in which certain benefits and profits are occurring often, and unacceptable costs are seldom.

#### Orchestrator-related protoforms

Orchestrator has three types of ILSs, as described in Sect. 4.2.3.

*Transaction perspective for an orchestrator:*

45 $$ qs_{2a} = \langle qf_{2a} ,transaction,f\left( {transaction} \right),f\left( {p_{2} .aben} \right)\rangle $$

46 $$ qs_{2b} = \langle qf_{2b} ,transaction,f\left( {transaction} \right),f\left( {p_{2} .acost} \right)\rangle $$

47 $$ qs_{2c} = \langle qf_{2c} ,transaction,f\left( {transaction} \right),f\left( {p_{2} .aben,p_{2} .acost} \right)\rangle $$

with $$qf_{2a} ,qf_{2c} \in \left\{ {ALL,ALMOST\;ALL, MOST} \right\} $$ and

$$ qf_{2b} \in \left\{ {NONE,ALMOST\;NONE,FEW} \right\} $$

*Single-aggregated customer perspective:*

48 $$ qs_{sa1a} = \langle qf_{sa1a} ,p_{1} ,\langle any Feature,all\;Values\rangle ,f\left( {p_{1} .aben} \right)\rangle $$

49 $$ qs_{sa1b} = \langle qf_{sa1b} ,p_{1} ,\langle any\;Feature,all\;Values\rangle ,f\left( {p_{1} .acost} \right)\rangle $$

50 $$ qs_{sa1c} = \langle qf_{sa1c} ,p_{1} ,\langle any\;Feature,all\;Values\rangle ,f\left( {p_{1} .aben,p_{1} .acost} \right)\rangle $$

with $$qf_{sa1a} ,qf_{sa1c} \in \left\{ {ALL,ALMOST\;ALL, MOST} \right\} $$ and

$$ qf_{sa1b} \in \left\{ {NONE,ALMOST\;NONE,FEW} \right\} $$

*Double-aggregated customer perspective:*

51 $$ qs_{da1a} = \langle qf_{da1a} ,p_{1} ,\langle any\;Feature,all\;Values\rangle , \langle qf_{da1d} ,transaction,f\left( {transaction} \right),f\left( {p_{1} .aben} \right)\rangle \rangle $$

52 $$ qs_{da1b} = \langle qf_{da1b} ,p_{1} ,\langle any\;Feature,all\;Values\rangle , \langle qf_{da1e} ,transaction,f\left( {transaction} \right),f\left( {p_{1} .acost} \right)\rangle \rangle $$

53 $$ qs_{da1c} = \langle qf_{da1c} ,p_{1} ,\langle any\;Feature,all\;Values\rangle , \langle qf_{da1f} ,transaction,f\left( {transaction} \right),f\left( {p_{1} .aben,p_{1} .acost} \right)\rangle \rangle $$

with $$qf_{da1a} ,qf_{da1c} ,qf_{da1d} ,qf_{da1e} ,qf_{da1f} \in \left\{ {ALL,ALMOST\;ALL, MOST} \right\} $$ and

$$ qf_{da1b} \in \left\{ {NONE,ALMOST\;NONE,FEW} \right\} $$

For the cost only one of the quantifiers is minority quantifier, i.e., describing amount smaller than half, as we want to avoid a double negation issue. A summary “*almost no* customers have *a few* transactions with *high cost”*, could indeed suggest that this high cost occurs often.

#### Other party-related protoforms

A party has one type of ILS according to specification in Sect. 4.2.4.

The party is $p_{k } for 3 \le k \le n, if b_{k}$.

*Transaction perspective for a party:*

544 $$ qs_{ka} = \langle qf_{ka} ,transaction,f\left( {transaction} \right),f\left( {p_{k} .aben} \right)\rangle $$

55 $$ qs_{kb} = \langle qf_{kb} ,transaction,f\left( {transaction} \right),f\left( {p_{k} .acost} \right)\rangle $$

56 $$ qs_{kc} = \langle qf_{kc} ,transaction,f\left( {transaction} \right),f\left( {p_{k} .aben,p_{k} .acost} \right)\rangle $$

with $$qf_{ka} ,qf_{kc} \in \left\{ {ALL,ALMOST\;ALL, MOST} \right\} $$ and

$$ qf_{kb} \in \left\{ {NONE,ALMOST\;NONE,FEW} \right\} $$

#### Network perspective

Network perspective allows to generate summaries over a set of parties, as introduced in Sect. 4.3.5:

*Single-aggregated party *perspective*:*

57 $$ qs_{saka} = \langle qf_{saka} ,p_{1} ,\langle any\;Feature,all\;Values\rangle ,f\left( {p_{k} .aben} \right)\rangle $$

58 $$ qs_{sakb} = \langle qf_{sakb} ,p_{1} ,\langle any\;Feature,all\;Values\rangle ,f\left( {p_{k} .acost} \right)\rangle $$

59 $$ qs_{sakc} = \langle qf_{sakc} ,p_{1} ,\langle any\;Feature,all\;Values\rangle ,f\left( {p_{k} .aben,p_{k} .acost} \right)\rangle $$

with $$qf_{saka} ,qf_{sakc} \in \left\{ {ALL,ALMOST\;ALL, MOST} \right\} $$ and

$$ qf_{sakb} \in \left\{ {NONE,ALMOST\;NONE,FEW} \right\} $$

*Double-aggregated party perspective:*

60 $$ qs_{daka} = \langle qf_{daka} ,p_{k} ,\langle any\;Feature,all\;Values\rangle , \langle qf_{dakd} ,transaction,f\left( {transaction} \right),f\left( {p_{k} .aben} \right)\rangle \rangle $$

61 $$ qs_{dakb} = \langle qf_{dakb} ,p_{k} ,\langle any\;Feature,all\;Values\rangle , \langle qf_{dake} ,transaction,f\left( {transaction} \right),f\left( {p_{k} .acost} \right)\rangle \rangle $$

62 $$ qs_{dakc} = \langle qf_{dakc} ,p_{k} ,\langle any\;Feature,all\;Values\rangle , \langle qf_{dakf} ,transaction,f\left( {transaction} \right),f\left( {p_{k} .aben,p_{k} .acost} \right)\rangle \rangle $$

with $$qf_{daka} ,qf_{dakc} ,qf_{dakd} ,qf_{dake} ,qf_{dakf} \in \left\{ {ALL,ALMOST\;ALL, MOST} \right\} $$ and $$qf_{dakb} \in \left\{ {NONE,ALMOST\;NONE,FEW} \right\}$$.
